# Endolysosome-targeted nanoparticle delivery of antiviral therapy for coronavirus infections

**DOI:** 10.26508/lsa.202403182

**Published:** 2025-02-03

**Authors:** Anton Petcherski, Brett M Tingley, Andrew Martin, Sarah Adams, Alexandra J Brownstein, Ross A Steinberg, Byourak Shabane, Jennifer Ngo, Corey Osto, Gustavo Garcia, Michaela Veliova, Vaithilingaraja Arumugaswami, Aaron H Colby, Orian S Shirihai, Mark W Grinstaff

**Affiliations:** 1 Department of Medicine, Division of Endocrinology, David Geffen School of Medicine, University of California Los Angeles, Los Angeles, CA, USA; 2 Department of Biomedical Engineering, Boston University , Boston, MA, USA; 3 Molecular Cellular Integrative Physiology, University of California Los Angeles, Los Angeles, CA, USA; 4 Department of Molecular and Medical Pharmacology, David Geffen School of Medicine, University of California Los Angeles, Los Angeles, CA, USA; 5 Department of Chemistry, Boston University , Boston, MA, USA

## Abstract

SARS-CoV-2 can infect cells through endocytic uptake, a process that is targeted by inhibition of lysosomal proteases. However, clinically this approach to treat viral infections has afforded mixed results, with some studies detailing an oral regimen of hydroxychloroquine accompanied by significant off-target toxicities. We rationalized that an organelle-targeted approach will avoid toxicity while increasing the concentration of the drug at the target. Here, we describe a lysosome-targeted, mefloquine-loaded poly(glycerol monostearate-co-ε-caprolactone) nanoparticle (MFQ-NP) for pulmonary delivery via inhalation. Mefloquine is a more effective inhibitor of viral endocytosis than hydroxychloroquine in cellular models of COVID-19. MFQ-NPs are less toxic than molecular mefloquine, are 100–150 nm in diameter, and possess a negative surface charge, which facilitates uptake via endocytosis allowing inhibition of lysosomal proteases. MFQ-NPs inhibit coronavirus infection in mouse MHV-A59 and human OC43 coronavirus model systems and inhibit SARS-CoV-2 WA1 and its Omicron variant in a human lung epithelium model. Organelle-targeted delivery is an effective means to inhibit viral infection.

## Abstract (precis)

Lysosome-targeting nanoparticles, loaded with mefloquine, inhibit coronavirus infection in mouse MHV-A59 and human OC43 coronavirus model systems, as well as SARS-CoV-2 WA1 and its Omicron variant in a human lung epithelium model.

## Introduction

To date, severe acute respiratory syndrome coronavirus 2 (SARS-CoV-2) has infected over 763 million individuals resulting in over 6.9 million deaths globally, causing significant harm to public health and resulting in a sizable humanitarian and socioeconomic burden (Nicola et al, 2020; WHO, 2023). To combat this crisis, Pfizer and Moderna, among others, developed highly efficacious vaccines (BNT162b2 and mRNA-1273, respectively) at unprecedented speeds, which display >94% efficacy in preventing COVID-19 illness, including severe disease (Polack et al, 2020; Baden et al, 2021). Despite rapid development and distribution of vaccines, the virus continues to gain genetic mutations in regions that have been targeted by recently developed prophylactics and treatments. Consequently, neutralization-resistant SARS-CoV-2 variants continue to spike worldwide case numbers and mortality (Dong et al, 2020; Garcia-Beltran et al, 2021).

Concurrently with vaccine development, multiple clinically approved drugs were rapidly repurposed to treat COVID-19 patients (Serafin et al, 2020). These include antimalarial agents such as chloroquine/hydroxychloroquine (CQ/HCQ), protease inhibitors such as lopinavir/ritonavir (LPV/RTV), and viral transcription inhibitors such as remdesivir (RDV). Despite encouraging in vitro reports, the clinical use of CQ/HCQ and LPV/RTV for patients with COVID-19 resulted in minimal or no clinical benefit over the standard of care (Cao et al, 2020; RECOVERY Collaborative Group, 2020; RECOVERY Collaborative Group et al, 2020; Patel et al, 2021; WHO Solidarity Trial Consortium et al, 2021). Viral transcription inhibitors such as RDV and other RNA-dependent RNA polymerase (RdRp) inhibitors block the viral transcription machinery from proceeding by inhibiting nucleotide incorporation into the viral mRNA (Malone et al, 2022; Shannon et al, 2022). Clinical trials evaluating RDV afforded mixed outcomes wherein RDV was superior to placebo in shortening the time to recovery in adults who were hospitalized with mild-to-severe COVID-19 in three randomized controlled clinical trials (Beigel et al, 2020; Goldman et al, 2020; Spinner et al, 2020) but failed to demonstrate improved clinical outcomes as indicated by mortality rates, initiation of ventilation, and total duration of hospital stay in both the WHO Solidarity trial and the DisCoVeRy trial (Ader et al, 2022; WHO, 2023). Emerging evidence suggests that RDV improves clinical outcomes, but only if administered within an early time window after infection (Gottlieb et al, 2022; Heil & Kottilil, 2022).

In addition to the early use of repurposed drugs, other small molecule viral inhibitors were developed such as nirmatrelvir (Pfizer, Paxlovid) and molnupiravir (Merck), which received Food and Drug Administration (FDA) approval a year after RDV. Nirmatrelvir is an orally bioavailable viral 3CL^pro^ inhibitor used in combination with ritonavir to slow the metabolism of the drug (Owen et al, 2021). In a randomized controlled trial for unvaccinated, non-hospitalized adults at high risk of progression to severe COVID-19, Paxlovid decreased the risk of progression to severe symptoms by 89% over placebo as determined by hospitalization rate and mortality (Hammond et al, 2022). More recently, Pfizer terminated the EPIC-SR trial (NCT05011513) as Paxlovid displayed no clinical benefit over placebo with regard to COVID-19 symptom relief in non-hospitalized symptomatic adults who are at low risk of progressing to severe illness. In addition, further in vitro testing in both a live SARS-CoV-2 and vesicular stomatitis virus (VSV)–based pseudovirus model suggests that selective pressure may lead to 3CL^pro^ mutations conferring nirmatrelvir resistance to new viral mutants (Jochmans et al, 2022 *Preprint*; Heilmann et al, 2023). Molnupiravir is a prodrug nucleoside analog, which causes the accumulation of significant point mutations in replicated viral transcripts (Kabinger et al, 2021). In a randomized controlled trial for non-hospitalized, unvaccinated adults with mild-to-moderate COVID-19 symptoms, early treatment (within 5 d of symptom onset) with molnupiravir resulted in a significant reduction in risk of hospitalization and mortality (Jayk Bernal et al, 2022). However, there is a growing concern that molnupiravir, especially when administered at subtherapeutic doses, may result in the creation of more virulent SARS-CoV-2 mutants (Agostini et al, 2019). The discrepancy between experimental results and clinical outcomes for COVID-19 candidates and the potential for neutralization-resistant mutants, or creation of more virulent strains from emerging COVID-19 therapies, necessitate the development of new strategies, therapeutics, and prophylactics against COVID-19, as well as delivery systems to target the virus or host cell while minimizing off-target toxicity.

There are several small molecule drugs that were also repurposed for the treatment of coronaviruses and indicated efficacy in preclinical models; however, they have not gained as much traction as CQ/HCQ and lopinavir. Nitazoxanide is a member of the thiazolide drug class, which are broad-spectrum anti-infection drugs, and shows early promise in both in vitro and small-scale in vivo trials (Mahmoud et al, 2020; Wang et al, 2020; Blum et al, 2021). The antiviral mechanism of action is not fully characterized, although reports suggest that nitazoxanide may target multiple stages of the SARS-CoV-2 life cycle, including endocytosis and membrane fusion, viral genome synthesis and viral protein processing, and the late-stage inflammatory response (Lokhande & Devarajan, 2021). Sulfadoxine belongs to a class of drugs known to interrupt the synthesis of folic acid. Several in vitro reports demonstrate anti-SARS-CoV-2 activity with sulfadoxine, although it is unclear whether folic acid synthesis plays a role in viral replication or whether sulfadoxine has a novel unknown inhibitory action (Arshad et al, 2020; Touret et al, 2020).

Mefloquine (MFQ), a 4-quinolinemethanol similar in structure to CQ/HCQ, shows improved activity against SARS-CoV-2 over CQ/HCQ (Shionoya et al, 2021; Sacramento et al, 2022). CQ/HCQ blocks SARS-CoV-2 entry only in cells lacking transmembrane serine protease 2 (TMPRSS2), whereas the expression of TMPRSS2 significantly reduces the antiviral activity of CQ/HCQ (Hoffmann et al, 2020; Ou et al, 2021). Existing literature suggests that mefloquine inhibits viral entry after viral attachment to the target cell (Shionoya et al, 2021; Sacramento et al, 2022). The exact mechanism is unknown; however, mefloquine may be (1) inhibiting viral membrane fusion with the cell membrane or endolysosomal membrane, (2) inhibiting proteases responsible for processing SARS-CoV-2 S protein and exposing the fusion peptide, (3) modulating expression levels of angiotensin converting enzyme 2 (ACE2), transmembrane protease, serine 2 (TMPRSS2), and/or cathepsin L (CTSL), or (4) promoting exocytosis of SARS-CoV-2 particles after uptake. Unlike CQ/HCQ, MFQ reduces viral load in clinically relevant cell lines, including Calu-3 and Vero E6/TMPRSS2 cells, which express both ACE2 and TMPRSS2 (Shionoya et al, 2021; Sacramento et al, 2022). Thus, MFQ is more broadly active against coronaviruses as compared to CQ/HCQ, and this dependence on the lack of TMPRSS2 may explain the discrepancy between in vitro and in vivo results reported using CQ/HCQ for the treatment of SARS-CoV-2.

Pharmacokinetic (PK) modeling and analysis further suggest that CQ/HCQ does not achieve therapeutic anti-SARS-CoV-2 concentrations in vivo when delivered orally (McLachlan et al, 1993; Liu et al, 2020; Touret et al, 2020). Moreover, multiple clinical trials assessing CQ/HCQ for the treatment of hospitalized patients with COVID-19 displayed an increased risk of drug-induced cardiac toxicities (Tleyjeh et al, 2021). Similar PK modeling of oral MFQ dosing predicts that plasma concentrations above the target EC_90_ can be achieved only with high doses over multiple days (e.g., 450 mg TID or 350 mg QID for 3 d), which likely leads to off-target effects (Karbwang & White, 1990; Shionoya et al, 2021; Sacramento et al, 2022). In fact, the prophylactic use of mefloquine for malaria prevention is known to cause neurotoxicity/neurological adverse events (e.g., abnormal dreams, insomnia, anxiety, depressed mood, nausea, dizziness, and chronic central nervous system toxicity syndrome); however, the mechanisms underlying its neurotoxic effects are poorly understood (McCarthy, 2015; Martins et al, 2021).

The non-specific delivery route for these small molecules (e.g., oral or intravenous) results in the administration of high doses with low drug accumulation in the target tissue (i.e., the lungs). As many of these small molecules are acutely cytotoxic beyond their therapeutic window, these high doses also lead to significant off-target effects in tissues not infected with virus. To address the unmet need for a potent antiviral treatment for coronaviruses that locally targets the pulmonary system, we describe nanoparticles based on biocompatible components and loaded with chloroquine, mefloquine, sulfadoxine, or nitazoxanide. The particles are composed of poly(glycerol monostearate-co-ε-caprolactone) (PGC-C18), and of these four payloads, mefloquine-loaded nanoparticles exhibit the strongest inhibitory effect on coronavirus infection. Herein, we report on negatively charged PGC-C18 nanoparticles (NPs) of 100–150 nm in diameter that physically entrap MFQ. MFQ-loaded NPs (MFQ-NPs) are rapidly taken up by cells, localize to endolysosomal compartments, and decrease protease activity. MFQ-NPs inhibit coronavirus infection in mouse MHV-A59 and human coronavirus OC43 model systems and inhibit SARS-CoV-2 WT and Omicron variant infection in a human lung epithelium cell line model.

## Results

### PGC-C18 nanoparticle formation and characterization studies

**Figure 1.**
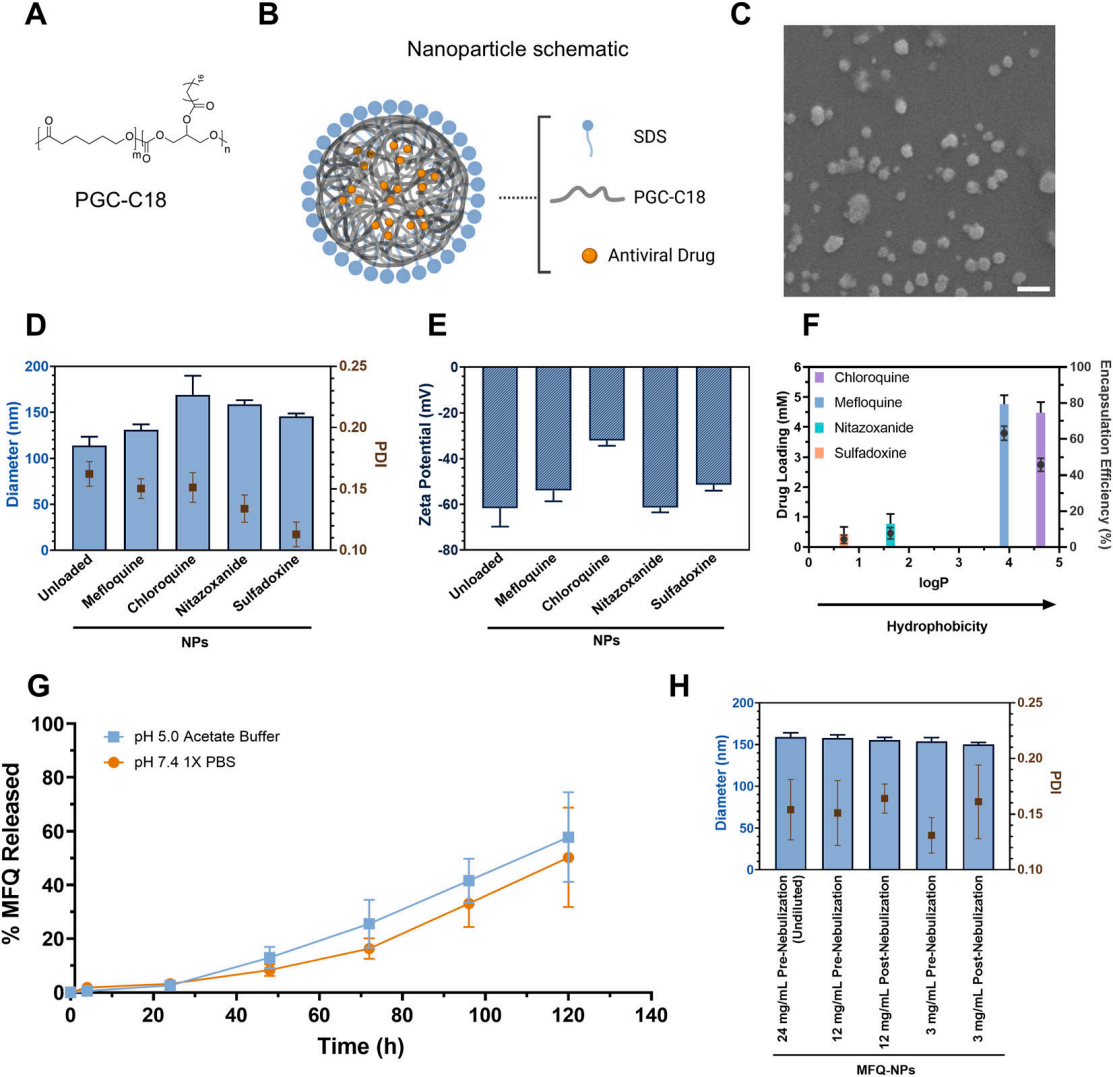
Formulation of novel PGC-C18 nanoparticles. **(A)** Chemical structure of poly(1,3-glycerol monostearate-co-ε-caprolactone) (PGC-C18). **(B)** Schematic structure of PGC nanoparticle containing sodium dodecyl sulfate surfactant and antiviral drug payload. **(C)** Electron micrographs of unloaded PGC-NPs demonstrate sizes around 100 nm and round morphology. Scale bar = 200 nm. **(D)** NP size and polydispersity measurements using dynamic light scattering (DLS) confirm nanoparticle sizes around 100–150 nm for unloaded and small molecule–loaded NPs. **(E)** Charge measurement of NPs using DLS. **(F)** Encapsulation efficiency of drug compounds in NPs as measured by high-performance liquid chromatography (HPLC). Drug loading concentrations are graphed as colored bars and measured on the left y-axis. Compound encapsulation efficiencies are graphed as dots (●) and measured on the right y-axis. **(G)** Mefloquine release from mefloquine-loaded NPs (MFQ-NPs) over 5 d in pH 7.4 and pH 5.0 release buffer. Release is plotted as a % of drug released relative to initial drug loading at day 0. **(H)** Size and polydispersity measurements by DLS confirm nanoparticle stability after nebulization with Aerogen Solo. All experiments in (A, B, C, D, E, F, G) represent N = 3–5 independent NP batches. All data are displayed as means ± SD. Source data are available for this figure.

**Figure S1.**
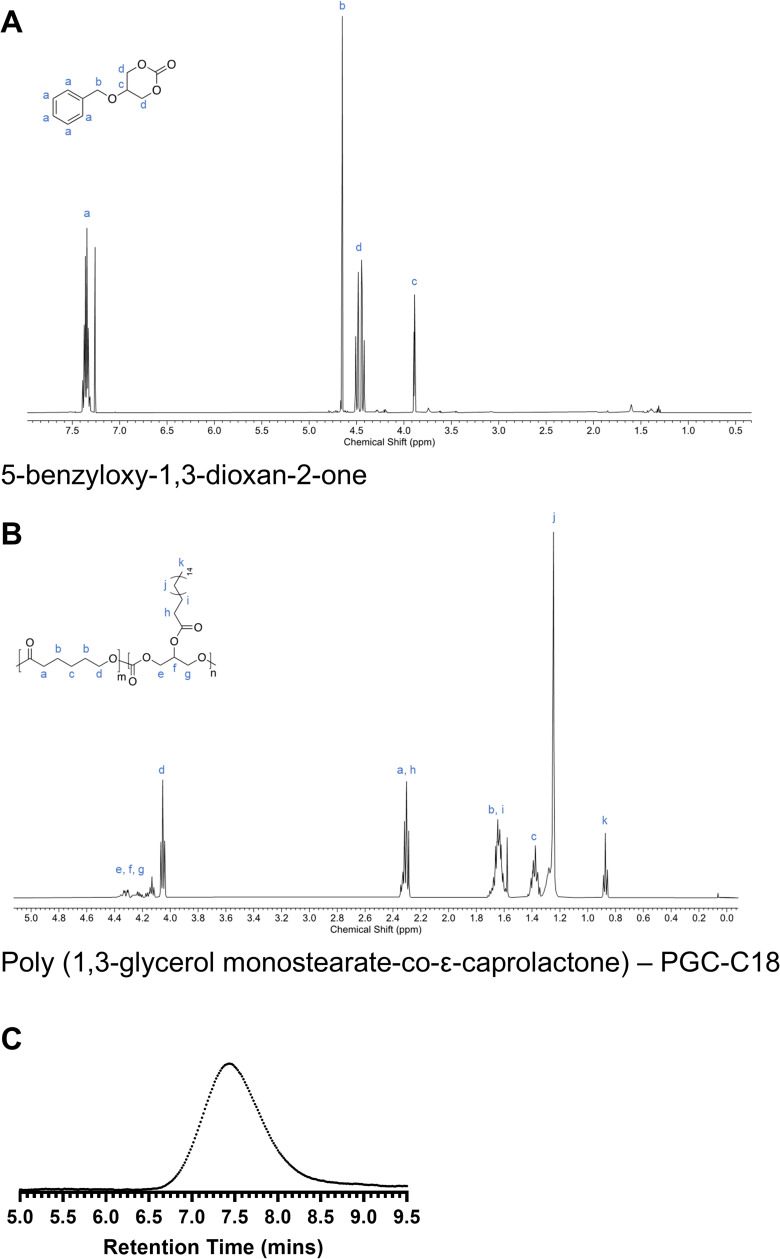
Characterization of poly(1,3-glycerol monostearate-co-ε-caprolactone) (PGC-C18). **(A)** ^1^H NMR (500 MHz, CDCl_3_) spectrum of 5-benzyloxy-1,3-dioxan-2-one monomer. **(B)** ^1^H NMR (500 MHz, CDCl_3_) spectrum of poly(1,3-glycerol monostearate-co-ε-caprolactone) (PGC-C18) using a 4:1 M ratio of ε-caprolactone to 5-benzyloxy-1,3-dioxan-2-one monomers. **(C)** THF GPC trace of PGC-C18. PGC-C18 molecular weight (M_n_) = 78,272 g/mol and dispersity (Đ) = 1.668 were determined based on polystyrene standards according to the refractive index (RI) detector. Source data are available for this figure.

First, we developed a method to prepare nanoparticles (NPs) with a spherical morphology of ∼100 nm. Polymeric NPs on this scale are amenable to delivery via inhalation and uptake by endocytosis (Geiser & Kreyling, 2010; Löndahl et al, 2014; Thorley et al, 2014). We selected poly(glycerol monostearate-co-ε-caprolactone), PGC-C18 (Fig 1A), as it is comprised of biocompatible degradation products of glycerol, CO_2_, stearate, and 6-hydroxyhexanoic acid, and we have a large-scale GMP-compatible synthetic method for producing it, which would be useful in speeding clinical translation (Kaplan et al, 2016). To synthesize the polymer, we copolymerized ε-caprolactone and 5-benzyloxy-1,3-dioxan-2-one monomers via ring-opening polymerization catalyzed by tin(II) 2-ethylhexanoate (Sn(Oct)_2_). We subsequently removed the benzyl-protecting groups of poly(5-benzyloxy-1,3-dioxan-2-one-co-ε-caprolactone) (PGC-Bn) via palladium-catalyzed hydrogenolysis and conjugated stearic acid to the newly exposed hydroxyl groups via dicyclohexylcarbodiimide (DCC) coupling. Post-coupling, we confirmed the polymer structure via ^1^H NMR (Fig S1A and B), and gel permeation chromatography (GPC) analysis reveals a molecular weight (M_n_) of 78,300 g/mol with narrow dispersity (Đ = 1.67) (Fig S1C).

**Figure S2.**
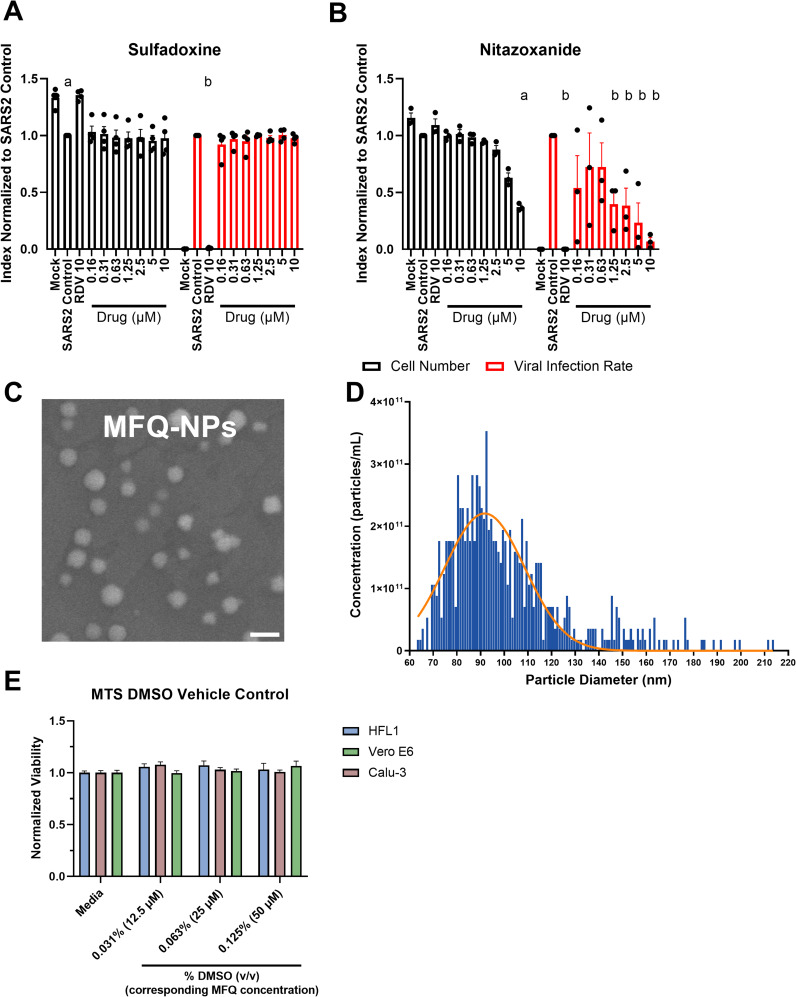
(Connected to Figs 1 and 2). **(A)** Quantification of cell numbers and fractions of infected cells in free molecular sulfadoxine–pretreated Vero E6 cells infected with SARS-CoV-2 WT-WA1. **(B)** Quantification of cell numbers and fractions of infected cells in free molecular nitazoxanide–pretreated Vero E6 cells infected with SARS-CoV-2 WT-WA1. **(C)** Electron micrograph of MFQ-NPs demonstrates consistent size and morphology. Scale Bar = 200 nm. **(D)** Size distribution of MFQ-NPs measured by tunable resistive pulse sensing (i.e., qNano). **(E)** Effects of different concentrations of DMSO (vehicle) treatments on HFL1, Vero E6, and Calu-3 cell viability measured by MTS assay. All biological replicates represent the mean of n = 3 technical replicates. All experiments represent N = 3–4 biological replicates. Sizing and dispersity data are displayed as means ± SD; all other data are displayed as means ± SEM. Source data are available for this figure.

We prepared drug-loaded NPs via the solvent evaporation method (Ekladious et al, 2017) using sodium dodecyl sulfate as the surfactant to stabilize the formation of spherical nanoparticles containing a core of PGC-C18 polymer encapsulating a hydrophobic drug payload (Fig 1B). SEM analysis of NP structure and size reveals spherical NPs of ∼100 nm diameter (Fig 1C). Quantitative analysis using dynamic light scattering (DLS) confirms the size range of 100–150 nm with a good uniformity reflected in a polydispersity index of <0.17 (Fig 1D). In addition, NP surface charge, as analyzed by DLS, is highly negative with a zeta potential of ≤−30 mV in unloaded and drug-loaded NPs (Fig 1E), which imparts NP stability (Ekladious et al, 2017). As PGC-C18 is hydrophobic, it favors the encapsulation of hydrophobic compounds with chloroquine and mefloquine (logP values of 4.63 and 3.9, respectively) encapsulating more effectively than the less hydrophobic sulfadoxine and nitazoxanide (logP of 0.7 and 1.63, respectively) (Fig 1F). Of the four drugs, mefloquine is the most effectively encapsulated compound with an encapsulation efficiency of ∼63%. In contrast, sulfadoxine and nitazoxanide lack sufficient encapsulation and exhibit negligible antiviral activity in vitro (Fig S2A and B). Therefore, we excluded both compounds from further studies. Likewise, chloroquine displays lower encapsulation efficiency and reduced in vitro activity against SARS-CoV-2 in cell models expressing TMPRSS2 compared with MFQ, and thus, we excluded it from further studies as well (Hoffmann et al, 2020; Sacramento et al, 2022). MFQ-loaded PGC-NPs (MFQ-NPs) exhibit controlled release over the span of 5 d in release buffer (i.e., 1X PBS [pH 7.4] or acetate buffer [pH 5.0] with 1 vol/vol% Tween-20) with 10–15% of loaded drug released after 48 h and 50–60% released at day 5 (Fig 1G). MFQ-NPs display a consistent spherical morphology and narrow size distribution (Fig S2C and D). Remarkably, MFQ-NPs retain size and dispersity after nebulization, further suggesting that this formulation is suitable for direct drug delivery into the lung (Fig 1H).

### Nanoparticles exhibit minimal in vitro cytotoxicity

**Figure 2.**
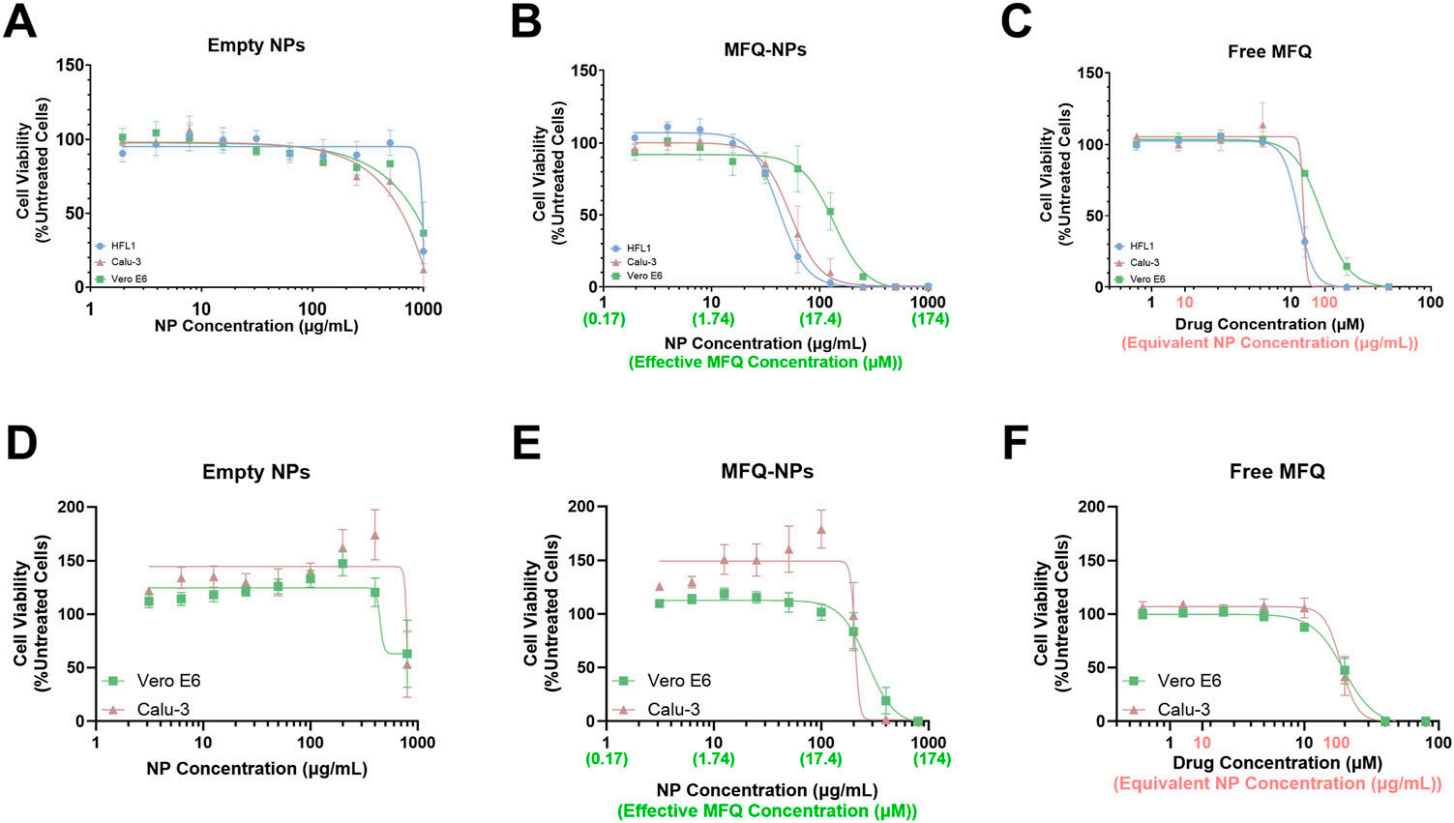
Cytotoxicity of PGC-C18 nanoparticles. **(A, B, C, D, E, F)** Cell viability measurements by MTS assay in HFL1, Calu-3, or Vero E6 cells treated with (A) unloaded PGC-NPs, (B) mefloquine-loaded NPs (MFQ-NPs), or (C) free molecular MFQ for 24 h or by CellTiter-Blue assay in Calu-3 or Vero E6 cells treated with (D) unloaded PGC-NPs, (E) MFQ-NPs, or (F) free MFQ for 72 h. All data are displayed as means ± SD. Each independent experiment represents the mean of n = 3 technical replicates. All experiments represent N = 3–6 biological replicates. Source data are available for this figure.

We evaluated NP cytotoxicity in vitro in HFL-1, Vero E6, and Calu-3 cell lines over 24 h via a tetrazolium-based MTS assay (Fig 2A–C). Vero E6 African Green Monkey kidney epithelial cells and Calu-3 human lung adenocarcinoma cells are widely used models of SARS-CoV-2 infection (Hoffmann et al, 2020; Sacramento et al, 2022), whereas HFL-1 cells are human embryonic lung fibroblasts. Unloaded (empty) NPs are relatively non-cytotoxic until dosed at high concentrations (>1 mg/ml). The IC_50_ values for MFQ-NPs are 42, 54, and 135 μg/ml (corresponding to ∼7.3, 9.4, and 23.5 μM of loaded MFQ) in HFL1, Calu-3, and Vero E6 cells, respectively. For reference, the IC_50_ values for MFQ in DMSO are 11.3, 12.3, and 16.6 μM for HFL1, Calu-3, and Vero E6 cells, respectively. The vehicle itself (i.e., DMSO) is not cytotoxic at equivalent concentrations without MFQ (Fig S2E). Notably, MFQ-NP treatments over 72 h show reduced cytotoxicity compared with the 24-h timepoints in Vero E6 and Calu-3 cells when assessed with the CellTiter-Blue assay (Fig 2D–F). For the 72-h timepoints, MFQ-NP IC_50_ values are 206 and 269 μg/ml (corresponding to ∼35.8 and 46.8 μM of loaded MFQ) for Calu-3 and Vero E6 cells, respectively. For MFQ in DMSO, the IC_50_ values are 18.8 and 19.6 μM for Calu-3 and Vero E6 cells, respectively, using this assay. These data suggest that MFQ-NPs mitigate MFQ cytotoxicity via slowing the release of MFQ into the cytosol compared with the “bolus” kinetics of free drug dosing.

### PGC-NPs target the lysosome

**Figure 3.**
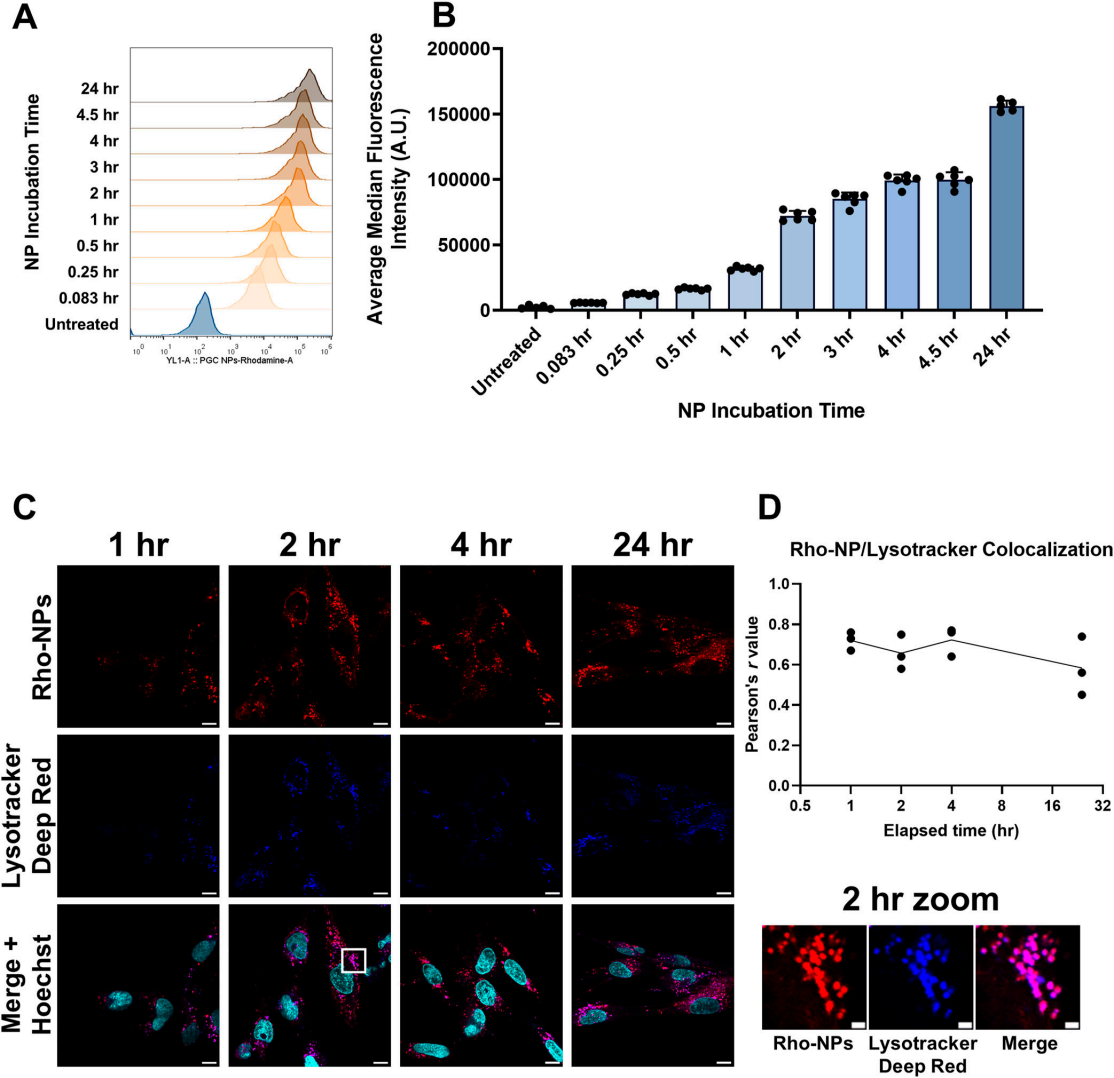
Nanoparticle uptake and localization. **(A, B)** Rho-NP uptake measured by flow cytometry in Calu-3 cells and displayed as (A) representative intensity histogram or (B) median intensity bar graph. **(C, D)** Confocal microscopy imaging of Rho-NPs and lysosomes labeled with LysoTracker Deep Red in HFL1 cells after 1, 2, 4, and 24 h of NP incubation demonstrates very high levels of colocalization (quantified as Pearson’s *r* coefficient in (D)). Scale bars = 10 μm; zoom-in box scale bars = 2 μm. All experiments represent N = 3 biological replicates, which represent the mean of at least n = 3 technical replicates. The intensity histogram displays a single representative cell population per timepoint. The intensity bar graph is displayed as medians ±SEM. The imaging experiment represents ∼50 cells per timepoint. Pearson’s coefficient was calculated from three individual images and is displayed as means ± SEM. Source data are available for this figure.

**Figure S3.**
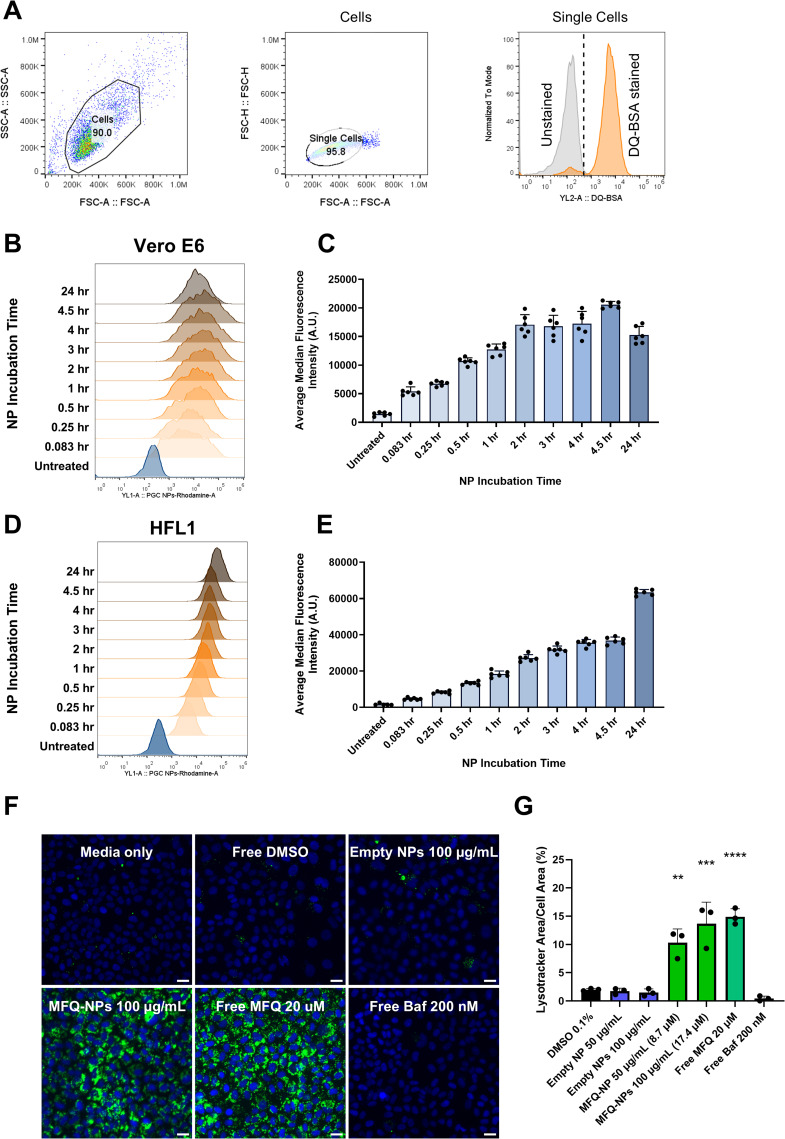
(Connected to Figs 3 and 4). **(A)** General pipeline outlining FlowJo gating of viable, single cells using forward and side scattering, resulting in histogram frequency distribution of individual cell fluorescence. Positive cell gating is based on unstained and untreated controls; representative unstained and stained controls from the DQ-Red BSA assay are shown. **(B, C)** Rho-NP uptake measured by flow cytometry in Vero E6 cells and displayed as (B) representative intensity histogram or (C) median intensity bar graph. **(D, E)** Rho-NP uptake measured by flow cytometry in HFL1 cells and displayed as (D) representative intensity histogram or (E) median intensity bar graph. **(F)** Representative images of Vero E6 cells treated with NPs ± MFQ (100 μg/ml), free MFQ (20 μM), bafilomycin A1 (200 nM), or control and stained with Hoechst 33342 (blue) and LysoTracker Green (green) to probe for lysosome accumulation. Scale bars = 20 μm. **(G)** Quantification of lysosomal accumulation. Statistical significance was determined by one-way ANOVA: **P < 0.01, ***P < 0.001, ****P < 0.0001 against untreated controls. All experiments represent N = 3 biological replicates, which represent the mean of at least n = 3 technical replicates. The intensity histogram displays a single representative cell population per timepoint. The intensity bar graph is displayed as medians ±SEM. All other experiments are displayed as means ± SEM. Source data are available for this figure.

To observe NP uptake, we formulated an NP containing covalently linked rhodamine B fluorophore (Rho-NPs) for analysis by flow cytometry and fluorescence microscopy. Flow cytometry reveals a rapid increase in rhodamine fluorescence after as little as 5 min of Rho-NP incubation with Calu-3, Vero E6, and HFL1 cells that steadily increases up to 24 h of Rho-NP incubation (Figs 3A and B and S3A–E).

As flow cytometry by itself is insufficient to demonstrate Rho-NP internalization rather than just surface adsorption, we performed confocal fluorescence microscopy to confirm localization of Rho-NPs in the endolysosomal system after cellular uptake (Fig 3C and D). Indeed, after 1 h of Rho-NP incubation, most of the Rho-NP fluorescence signal colocalizes with the lysosomal live-cell dye LysoTracker Deep Red (Pearson’s coefficient, *r* = 0.74). Rho-NP colocalization with LysoTracker is consistent over a period of 24 h (Fig 3C and D).

### MFQ-PGC-NPs inhibit lysosomal activity

**Figure 4.**
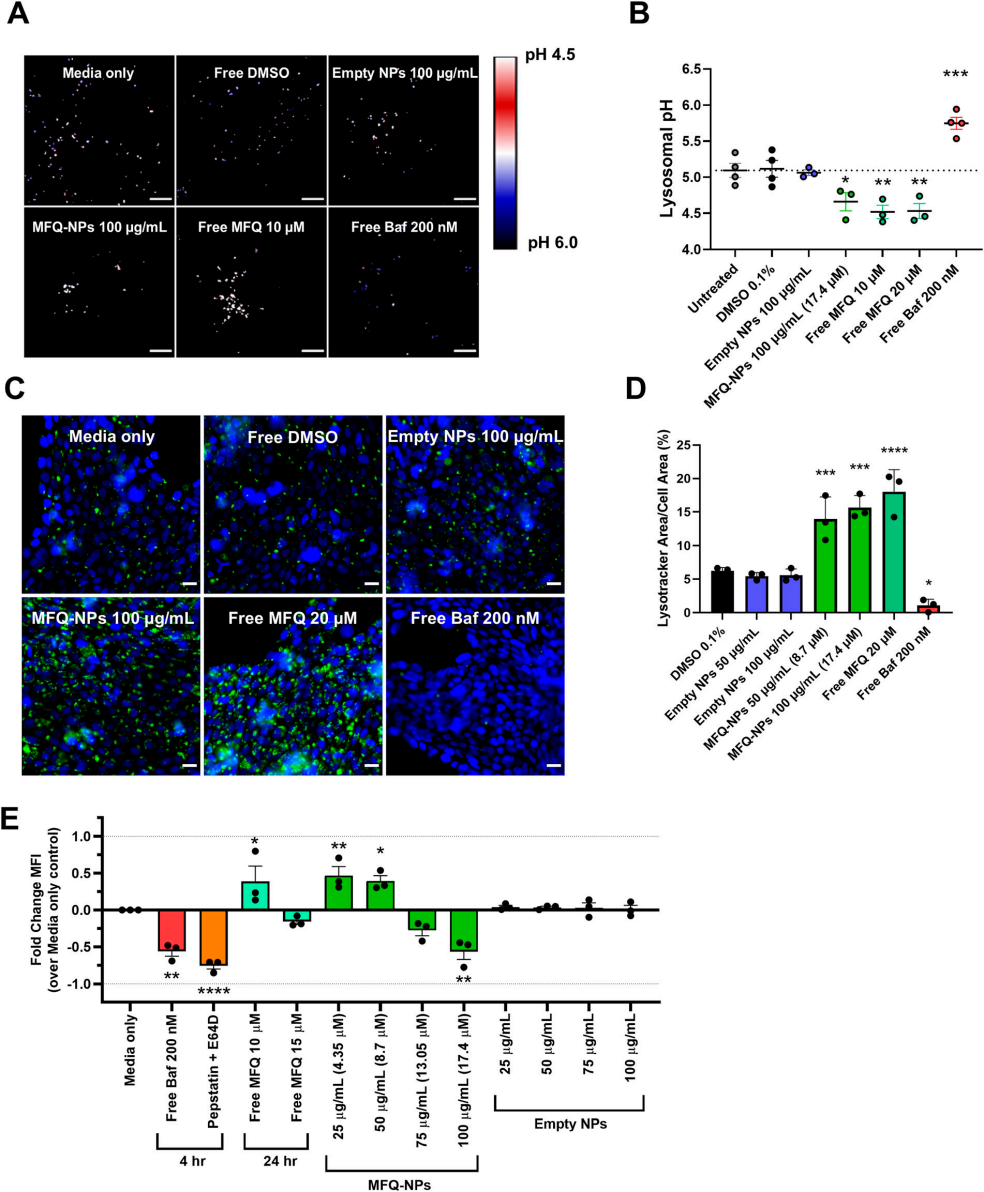
Nanoparticle effects on lysosomal pH and protease activity. **(A)** Representative confocal images of lysosomal pH measurements using LysoSensor Yellow/Blue dextran in Vero E6 cells treated with NP (±MFQ) 100 μg/ml, free MFQ 10 μM, bafilomycin 200 nM, or control. Scale bars = 10 μm. **(B)** Quantification of lysosomal pH. **(C)** Representative images of Calu-3 cells treated with NPs (±MFQ) 100 μg/ml, free MFQ 20 μM, bafilomycin 200 nM, or control and stained with Hoechst 33342 (blue) and LysoTracker Green (green) to probe for lysosome accumulation. Scale bars = 20 μm. **(D)** Quantification of lysosomal accumulation. **(E)** Quantification of lysosomal protease activity by DQ-Red BSA assay in Vero E6 cells treated with NPs (±MFQ), free MFQ, bafilomycin A1, pepstatin A + E64d, or controls at the indicated concentrations. Statistical significance was determined by one-way ANOVA: *P < 0.05, **P < 0.01, ***P < 0.001, ****P < 0.0001 against untreated controls. Each independent LysoSensor imaging experiment represents the mean of n = 10–20 individual images. All other biological replicates represent the mean of n = 3 technical replicates. All experiments represent N = 3 biological replicates. All data are displayed as means ± SEM. Source data are available for this figure.

Using Vero E6 cells, we evaluated changes in lysosomal pH with the ratiometric probe LysoSensor Yellow/Blue dextran. Free MFQ and MFQ-NPs do not increase lysosomal pH but, rather, induce further acidification (Fig 4A and B). Lysosomal pH of cells treated for 24 h with free MFQ or MFQ-NPs decreases from pH 5.1 to 4.4 (*P* < 0.05). In contrast, treatment for 2 h with 200 nM bafilomycin A1 increases lysosomal pH to 5.7 (*P* < 0.01). Unloaded NPs exert no effect on lysosomal pH. Although MFQ-NPs increase lysosomal acidity, this effect does not correlate with an increase in lysosomal function as free mefloquine and MFQ-NPs promote lysosomal accumulation in Calu-3 and Vero E6 cells (Figs 4C and D and S3F and G). Notably, 24-h treatments with free MFQ and MFQ-NPs afford a 2.5–3-fold increase in the amount of lysosomal staining area in Calu-3 cells (*P* < 0.001) and a 6.2–6.9-fold increase in Vero E6 cells (*P* < 0.0001). Bafilomycin A1 treatment strongly inhibits LysoTracker staining presumably by dissipating lysosomal pH. Free MFQ and MFQ-NP treatments, at concentrations above 15 μM, reduce lysosomal protease activity to the same degree as treatment with bafilomycin A1 or the lysosomal protease inhibitors pepstatin A and E64d by 57–75% (*P* < 0.01) (Fig 4E). Interestingly, MFQ concentrations below 10 μM increase lysosomal protease activity by up to 47% (*P* < 0.01), potentially because of the activation of compensatory lysosomal acidification. Unloaded NPs exhibit no effect on lysosomal protease activity.

### MFQ-PGC-NPs inhibit murine coronavirus MHV-A59 infection

**Figure S4.**
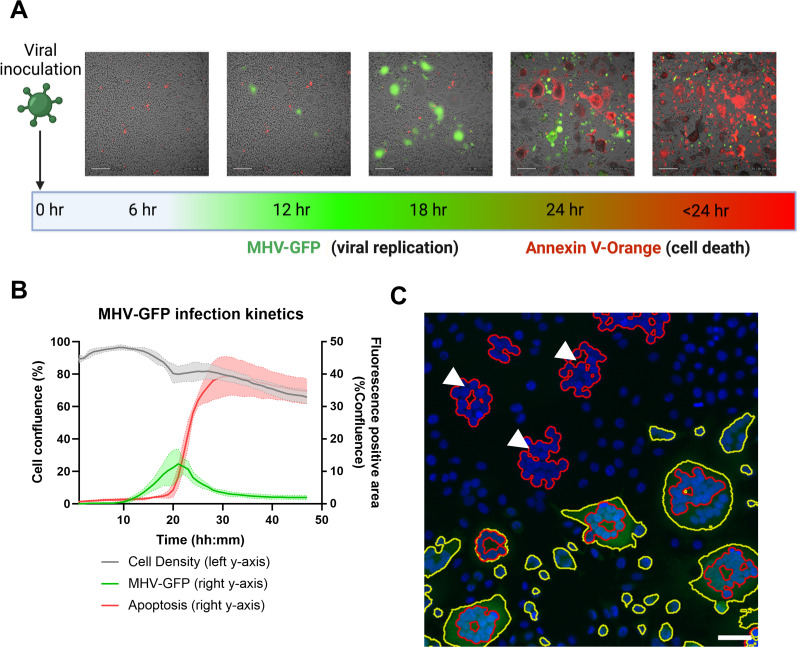
(Connected to Fig 5). **(A)** Timeline of viral inoculation, GFP expression, and apoptosis in MHV-GFP–infected L929 cells obtained by continuous image-based monitoring with a CytoSMART OmniFL analysis platform. Brightfield cell images are overlaid with GFP fluorescence (green) and Annexin V-Orange fluorescence (red). Scale bars = 200 μm. **(B)** Representative viral infection kinetic quantification over a 48-h infection period. **(C)** Representative example of syncytia (red outlines) recognized by CellProfiler analysis as clustered nuclei and GFP-positive area (yellow outlines) in fluorescence microscopy images obtained with the Operetta high-content imager. Nuclei (blue) are stained with DAPI, and MHV (green) is visualized by GFP expression. GFP-negative syncytia are marked with white arrowheads. Scale bar = 50 μm. CytoSMART Omni FL experiment biological replicates represent the mean of n = 3 technical replicates. Experiments represent N = 3 biological replicates. Source data are available for this figure.

**Figure 5.**
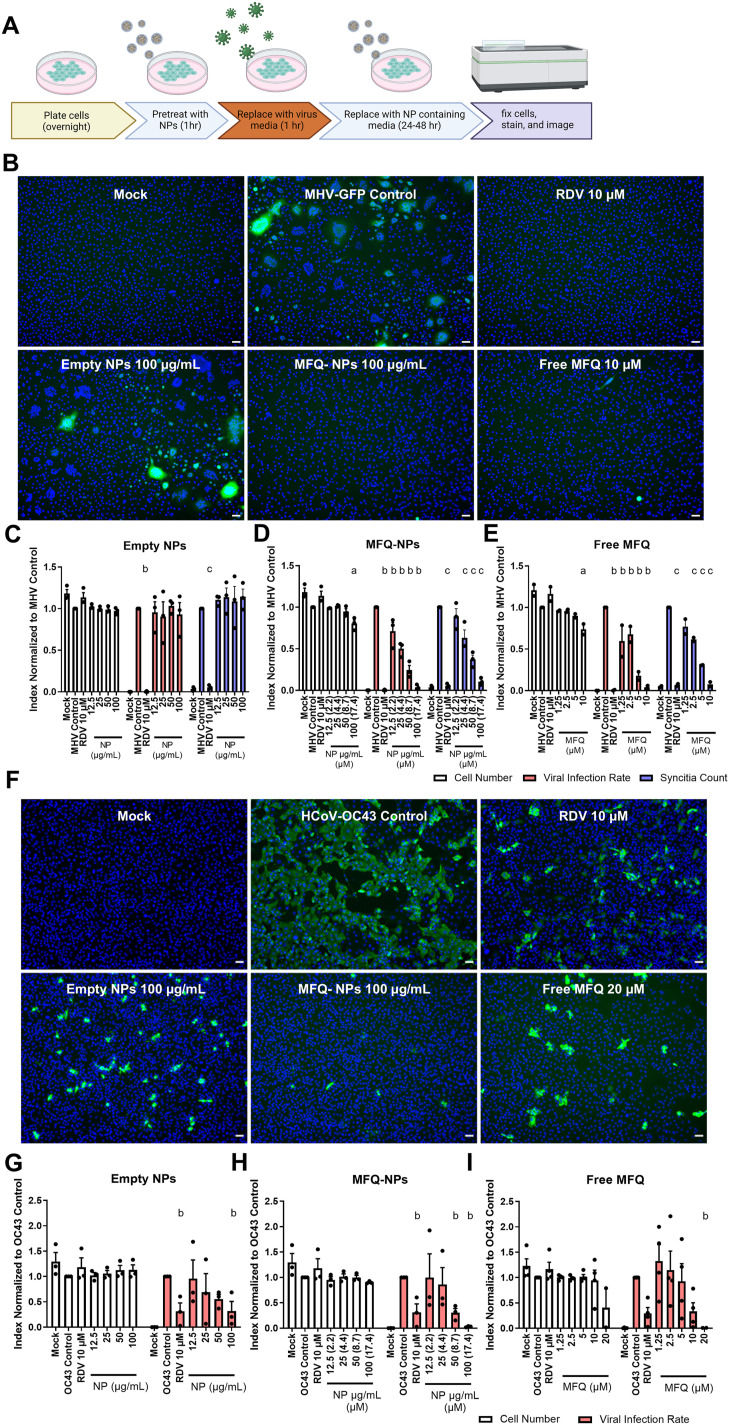
PGC-NPs loaded with MFQ are effective at inhibiting MHV and HCoV-OC43 infection. **(A)** Schematic describing the treatment and infection sequence of prophylactic NP–treated MHV-GFP or HCoV-OC43–infected cells. **(B)** Representative images of L929 cells pretreated with control, remdesivir (RDV, 10 μM), free MFQ (10 μM), or NPs (±MFQ) (100 μg/ml) and infected with MHV-GFP (MOI 0.1) for 24 h. Nuclei were stained with DAPI (blue), and virus-infected cells were visualized by GFP-positive signal (green) or syncytium formation. Scale bars = 50 μm. **(C, D, E)** Quantification of cell numbers, GFP area per image, and syncytia in unloaded NP (C)–, MFQ-NP (D)–, or free MFQ (E)–treated cells. Statistical significance was determined by two-way ANOVA: a, (at least) *P* < 0.05 against MHV-Control cell numbers; b, (at least) *P* < 0.05 against MHV-Control–infected cell area; c, *P* < 0.05 against MHV-Control syncytium count. **(F)** Representative images of Vero E6 cells pretreated with control, RDV (10 μM), free MFQ (20 μM), or NPs (±MFQ) (100 μg/ml) and infected with HCoV-OC43 (MOI 1) for 48 h. Nuclei were stained with DAPI (blue), and virus-infected cells were visualized by immunofluorescence staining against pan-coronavirus nucleocapsid (green). Scale bars = 50 μm. **(D, G, H, I)** Quantification of cell numbers and fractions of infected cells in empty NP (G)–, MFQ-NP (H)–, or free MFQ (I)–treated cells. Statistical significance was determined by two-way ANOVA: a, (at least) *P* < 0.05 against OC43-Control cell numbers; b, (at least) *P* < 0.05 against OC43-Control–infected cell fraction. All biological replicates represent the mean of n = 3 technical replicates. **(E)** Free MFQ–treated MHV-GFP (E) represents N = 2 biological replicates, and all other experiments represent N = 3 biological replicates. All data are displayed as means ± SEM. Source data are available for this figure.

Mouse hepatitis virus A59 (MHV-A59) is a beta coronavirus that infects mice displaying neuro-, hepato-, and pneumotropism depending on the route of infection (Cowley & Weiss, 2010). Like SARS-CoV-2, MHV-A59 displays multi-organ involvement and leads to more severe pneumonia in aged individuals (Ryu et al, 2021). L929 mouse fibroblasts infected with MHV-A59-GFP produce a lytic infection with extensive syncytium formation (Fig S4A–C). Preliminary studies using L929 cells indicated that MHV-A59-GFP infection, assessed by GFP positivity, peaked at 20–24 h post-infection, after which cell death, measured by Annexin V positivity, rapidly sets in (Fig S4A and B). Because dying cells rapidly lose their GFP+ signal, we included syncytium formation as an additional measure of viral infection frequency (Fig S4C). We adopted a preincubation protocol that allowed us to observe MFQ-NP effects on viral binding and uptake (Fig 5A). Preincubation with empty PGC-NPs exerts no preventive effect on MHV-A59-GFP viral infection (Fig 5B and C). In contrast, preincubation with MFQ-NPs and free MFQ reduces the amount of GFP+ cells and the incidence of syncytium formation in a dose-dependent manner (Fig 5B–E) by 29% at 12.5 μg/ml and up to 97% at the highest concentration of 100 μg/ml (*P* < 0.0001). At the highest tested concentrations (100 μg/ml for MFQ-NPs and 10 μM for free MFQ), a slight reduction in overall cell counts occurs compared with infected controls (∼20–27% reduction, *P* < 0.05). As a positive control, remdesivir completely inhibits viral replication at a concentration of 10 μM. Occasionally, individual cells treated with MFQ or MFQ-NPs still exhibit GFP-positive fluorescence; however, the infection did not spread in the culture (Fig 5B).

### MFQ-PGC-NPs inhibit human coronavirus OC43 infection

Like SARS-CoV-2, HCoV-OC43 is a beta coronavirus that causes respiratory tract infections in humans; however, HCoV-OC43 infections are generally mild cold-like symptoms. Vero E6 cells, infected with a high titer of HCoV-OC43 (MOI = 1), exhibit pronounced virus positivity after 48 h (Fig 5F, “virus-infected control”). Interestingly, remdesivir treatment results in an incomplete protection against viral infection at 10 μM, reducing viral infection rates by about 69% (*P* < 0.05). Surprisingly, empty NPs inhibit viral infection at the highest tested concentration of 100 μg/ml with inhibition similar in magnitude to remdesivir controls (68% reduction, *P* < 0.05) (Fig 5F and G). MFQ-NPs display a concentration-dependent inhibition of viral infection that is statistically significant at 50 μg/ml (70% reduction, *P* < 0.05) (Fig 5F–I) and reaches almost 100% at 100 μg/ml, whereas free MFQ inhibits viral replication at 10 μM but is only significant at 20 μM, at which concentration one observes substantial cytotoxicity.

### MFQ-PGC-NPs inhibit infection with SARS-CoV-2 WT-WA1 and Omicron BA.1 variants

**Figure 6.**
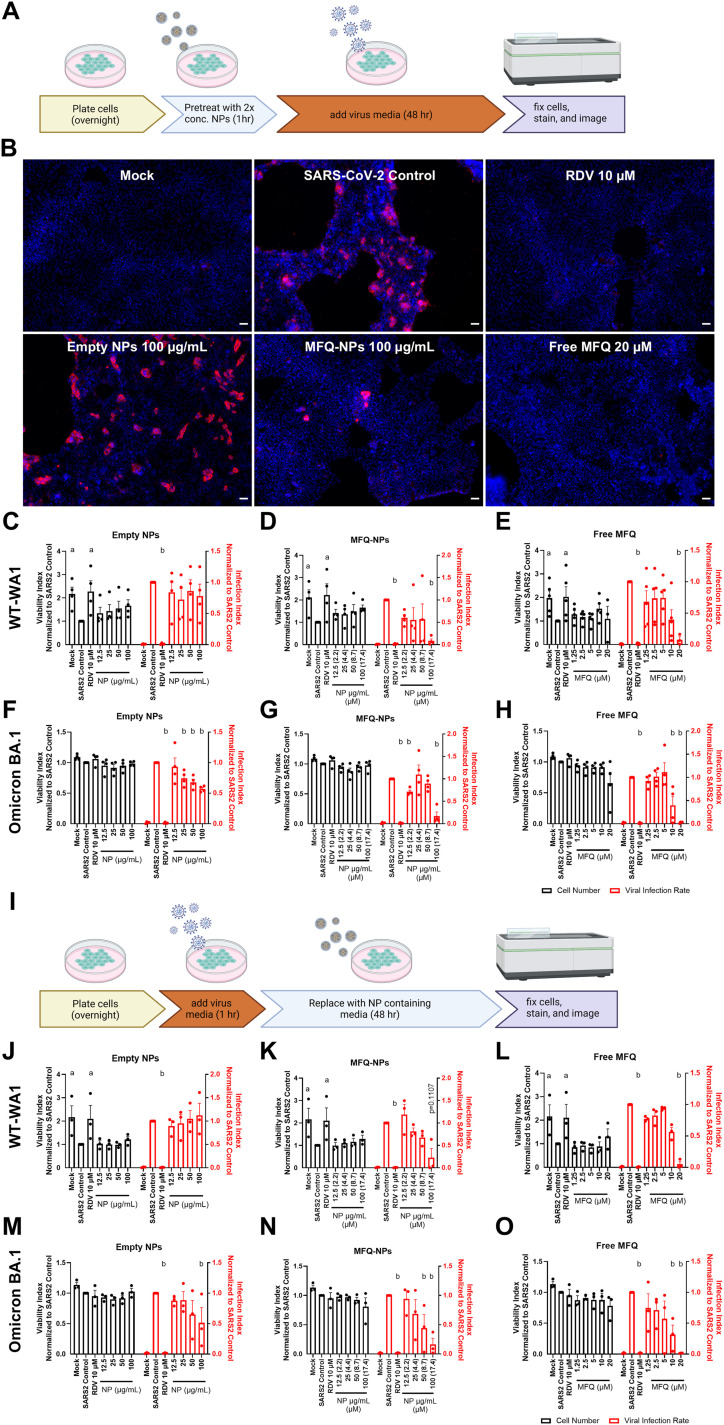
PGC-NPs loaded with MFQ are effective at inhibiting SARS-CoV-2 WT-WA1 and Omicron infection and replication. **(A)** Schematic describing the treatment and infection sequence of prophylactic NP–treated SARS-CoV-2–infected cells. **(B)** Representative images of Calu-3 cells pretreated with control, RDV (10 μM), free MFQ (20 μM), or NPs (±MFQ) (100 μg/ml) and infected with SARS-CoV-2 WT-WA1 (MOI 0.2) for 48 h. Nuclei were stained with DAPI (blue), and virus-infected cells were visualized by immunofluorescence staining against SARS-CoV-2 N protein (red). Scale bars = 50 μm. **(C, D, E)** Quantification of cell numbers and fractions of infected cells in unloaded NP (C)–, MFQ-NP (D)–, or free MFQ (E)–pretreated Calu-3 cells infected with SARS-CoV-2 WT-WA1. **(F, G, H)** Quantification of cell numbers and fractions of infected cells in unloaded NP (F)–, MFQ-NP (G)–, or free MFQ (H)–pretreated Calu-3 cells infected with SARS-CoV-2 Omicron BA.1. Statistical significance was determined by two-way ANOVA: a, (at least) *P* < 0.05 against SARS-CoV-2-Control cell numbers; b, (at least) *P* < 0.05 against SARS-CoV-2-Control–infected cell area. **(I)** Schematic describing the treatment and infection sequence of SARS-CoV-2–infected cells treated after infection. **(J, K, L)** Quantification of cell numbers and fractions of infected cells in unloaded NP (J)–, MFQ-NP (K)–, or free MFQ (L)–treated Calu-3 cells infected with SARS-CoV-2 WT-WA1. **(M, N, O)** Quantification of cell numbers and fractions of infected cells in unloaded NP (M)–, MFQ-NP (N)–, or free MFQ (O)–treated Calu-3 cells infected with SARS-CoV-2 Omicron BA.1. Statistical significance was determined by two-way ANOVA: a, (at least) *P* < 0.05 against SARS-CoV-2-Control cell numbers; b, (at least) *P* < 0.05 against SARS-CoV-2-Control–infected cell area. All biological replicates represent the mean of n = 3 technical replicates. All experiments represent N = 3–4 biological replicates. All data are displayed as means ± SEM. Source data are available for this figure.

**Figure S5.**
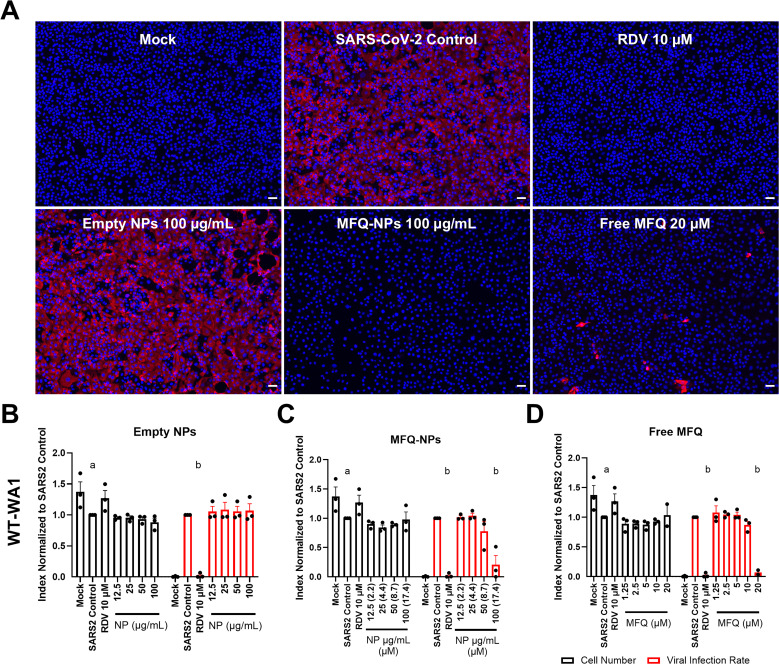
(Connected to Fig 6). **(A)** Representative images of Vero E6 cells pretreated with control, RDV (10 μM), free MFQ (20 μM), or NPs ± MFQ (100 μg/ml) and infected with SARS-CoV-2 WT-WA1 (MOI 0.1) for 48 h. Nuclei were stained with DAPI (blue), and virus-infected cells were visualized by immunofluorescence staining against SARS-CoV-2 N protein (red). Scale bars = 50 μm. **(B, C, D)** Quantification of cell numbers and fractions of infected cells in unloaded PGC-NP (B), MFQ-NP (C), or free MFQ (D)–pretreated Vero E6 cells infected with SARS-CoV-2 WT-WA1. Statistical significance was determined by two-way ANOVA: a, (at least) *P* < 0.05 against SARS-CoV-2-Control cell numbers; b, (at least) *P* < 0.05 against SARS-CoV-2-Control–infected cell area. All biological replicates represent the mean of n = 3 technical replicates. All experiments represent N = 3 biological replicates. Data are displayed as means ± SEM. Source data are available for this figure.

To assess MFQ-NP efficacy against the COVID-19 pandemic virus, SARS-CoV-2, we used two different cell lines: Vero E6 cells that do not express human TMPRSS2, therefore favoring SARS-CoV-2 infection by endocytosis; and Calu-3 human alveolar epithelial adenocarcinoma cells with a high expression of TMPRSS2, thus favoring SARS-CoV-2 spike cleavage and fusion at the plasma membrane. We initially adopted a similar preinfection treatment, which did not include washing off the viral inoculate (Fig 6A). In Vero cells, remdesivir (10 μM) effectively inhibits SARS-CoV-2 infection (98% reduction, *P* < 000.1) (Fig S5A and B), whereas empty PGC-NPs exhibit no effect, and MFQ-NPs reduce infection only at 100 μg/ml (79% reduction, *P* < 0.0001). Moreover, free MFQ reduces infection only at 20 μM (93% reduction, *P* < 0.001) without exhibiting significant toxicity (Fig S5A–D). In Calu-3 cells, MFQ-NP treatment at 100 μg/ml significantly reduces the number of SARS-CoV-2–positive cells (91%, *P* < 0.05), and similarly, strong inhibition was observed by free MFQ at 20 μM (92% reduction, *P* < 0.05) (Fig 6B–E). In addition to the ancestral SARS-CoV-2 variant, WT-WA1, we assessed MFQ-NP efficacy against Omicron BA.1. After preincubation, empty PGC-NPs exert a small inhibitory effect on Omicron infection at 25–100 μg/ml (26–43% reduction, *P* < 0.01–0.0001) (Fig 6F). MFQ-NPs significantly inhibit Omicron infection at 12.5 and 100 μg/ml (29% and 83% inhibition, *P* < 0.05 and <0.0001, respectively) (Fig 6G). Free MFQ significantly inhibits Omicron infection at 10 and 20 μM (60–97% inhibition, *P* < 0.001–0.0001) (Fig 6H).

### MFQ-PGC-NPs inhibit post-exposure spread of SARS-CoV-2 WT-WA1 and Omicron BA.1

The experimental design used to assess MFQ-NP efficacy under prophylactic treatment conditions cannot distinguish between decreased viral binding and endocytosis, and inhibition of viral replication. Thus, to study a potential inhibition of viral replication, we modified the protocol to include a 1-h incubation with SARS-CoV-2 inoculum before starting MFQ-NP treatments, which allows for viral attachment and uptake before the beginning of the treatment (Fig 6I). Unloaded PGC-NPs do not prevent SARS-CoV-2 WT-WA1 replication but exhibit a significant effect in Omicron-infected Calu-3 cells (49% reduction, *P* < 0.05) (Fig 6J and M). MFQ-NPs inhibit viral replication of the ancestral WT-WA1 variant to a non-significant degree (77% reduction, *P* = 0.11) and significantly reduce Omicron replication at 50 and 100 μg/ml (56% and 84% reduction, *P* < 0.01 and <0.001) (Fig 6K and N), which is similar to the result obtained with free MFQ at 10 and 20 μM (up to 98% reduction of Omicron-positive cells, *P* < 0.0001) (Fig 6L and O). These data suggest that MFQ-NPs inhibit viral uptake and replication.

### MFQ-NPs inhibit coronavirus infection in a post-attachment phase and alter the expression of proteins responsible for viral uptake

We next addressed which stage in the viral life cycle free MFQ and MFQ-NPs inhibit by performing viral attachment and entry assays and assessing the mRNA expression level of entry proteins after NP treatment.

**Figure S6.**
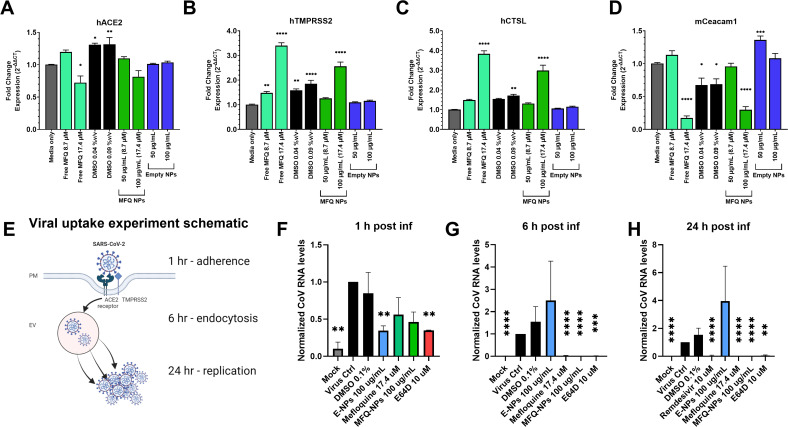
RNA expression level of essential proteins responsible for viral uptake and temporal NP effects on viral adherence and uptake. **(A, B, C, D)** ACE2 expression in Calu-3 cells, (B) TMPRSS2 expression in Calu-3 cells, (C) cathepsin L expression in Calu-3, and (D) CEACAM1 expression in L929 cells after 48-h treatment with unloaded PGC-NPs, MFQ-NPs, free MFQ solubilized in DMSO, DMSO as a vehicle control, or no-treatment control. **(E)** Schematic representation of the experimental timeline of SARS-CoV-2 uptake into the cell. **(F, G, H)** Viral RNA levels after (F) 1 h, (G) 6 h, and (H) 24 h of viral incubation in Vero cells pretreated with empty PGC-NPs, MFQ-NPs, free MFQ solubilized in DMSO, DMSO as a vehicle control, or no-treatment control. All biological replicates represent the mean of n = 3 technical replicates. All experiments represent N = 3–5 biological replicates using three independent PGC-NP or MFQ-NP batches. **(A, B, C, D, F, G, H)** Data are displayed as means ± SD (A, B, C, D) or SEM (F, G, H). Statistical significance was determined by one-way ANOVA: *P < 0.05, **P < 0.01, ***P < 0.001, ****P < 0.0001 against untreated controls; or a mixed-effects ANOVA: *P < 0.05, **P < 0.01, ***P < 0.001, ****P < 0.0001 against untreated controls. Source data are available for this figure.

In the case of SARS-CoV-2, the spike (S) protein binds to the ACE2 receptor on the surface of the cell. After attachment, the viral particle is subjected to S protein cleavage by host cell proteases, either TMPRSS2 on the cell membrane or CTSL after endosomal uptake (Matsuyama et al, 2010; Glowacka et al, 2011; Ou et al, 2021; Zhao et al, 2021). S protein cleavage initiates fusion pore formation between the viral membrane and the plasma membrane or endolysosomal membrane leading to viral RNA release into the cytoplasm and viral replication. Previous work (Brufsky, 2020; Shionoya et al, 2021; Yao et al, 2022) suggests that CQ, HCQ, and MFQ may impact the expression of target cell entry proteins or entry protein glycosylation. As MFQ is very similar in structure to these compounds, we sought to determine whether MFQ alters the expression of entry proteins responsible for SARS-CoV-2 uptake (e.g., ACE2, TMPRSS2, and CTSL) and MHV uptake (e.g., CEACAM1). To this end, Calu-3 or L929 cells were plated and allowed to adhere overnight. The next day, cell culture media were replaced with media containing free MFQ, MFQ-NPs, empty NPs, or DMSO control, or untreated media for 48 h. After treatment, RNA was isolated, and expression was measured using RT–qPCR for hACE2, hTMPRSS2, and hCTSL in Calu-3 cells and mCEACAM1 in L929 cells (Fig S6A–D). We observe a dosage-dependent fold increase in the expression of both hTMPRSS2 and hCTSL in Calu-3 cells with free MFQ and MFQ-NPs. Conversely, the expression of hACE2 reduces after treatment with free MFQ and MFQ-NPs. Unlike treatments containing high-dose MFQ, all other treatments (e.g., empty NPs and DMSO controls) display relatively similar expression levels of entry protein transcripts compared with media-only controls. In L929, the expression of mCEACAM1 significantly decreases after high-dose MFQ treatment in free drug or NP formulations. However, empty NPs do not reduce the expression of mCEACAM1 relative to untreated control, and DMSO-treated groups significantly reduced expression but not to an equal extent as the high-dose (i.e., 17.4 μM) free MFQ and MFQ-NPs.

To assess whether MFQ or MFQ-NPs inhibit viral attachment, we pretreated Vero E6 cells with varying concentrations of NPs or compounds for 1 h. After pretreatment, cells were exposed to SARS-CoV-2 for 1 h at 4°C to allow for viral attachment but not entry and replication (Fig S6E). After washing away unattached virus and NPs or compounds, we extracted and quantified the viral RNA attached to the cell surface. Cell membrane–associated SARS-CoV-2 RNA non-significantly reduces after treatment with free MFQ or MFQ-NPs (Fig S6F). Surprisingly, empty NPs (*P* = 0.007) and E64d (*P* = 0.007) significantly inhibit viral attachment.

To evaluate whether MFQ or MFQ-NPs inhibit viral endocytosis and membrane fusion (post-attachment phase), we similarly pretreated Vero E6 cells with varying concentrations of NPs or compounds for 1 h. After pretreatment, cells were exposed to SARS-CoV-2 for 1 h at 37°C to allow for viral attachment. After washing away unattached virus, we incubated the cells in growth media containing the NPs or compounds for 6 and 24 h, respectively, before extracting and quantifying the internalized viral RNA. After only 6 h of incubation, post-attachment viral RNA significantly diminishes after treatment with free MFQ (*P* < 0.0001), MFQ-NPs (*P* < 0.0001), and lysosomal protease inhibitor E64d (*P* = 0.0002) (Fig S6G). However, empty NPs exert no effect on viral RNA levels, suggesting that MFQ is responsible for the reduction in viral RNA post-attachment. After 24 h of incubation, viral RNA significantly reduces to levels comparable to remdesivir after treatment with free MFQ (*P* < 0.0001), MFQ-NPs (*P* < 0.0001), and E64d (*P* = 0.0055). Empty NPs similarly fail to reduce viral RNA levels after a 24-h incubation (Fig S6H).

## Discussion

The COVID-19 pandemic highlights the capacity of a sudden infectious disease to cause a long-lasting impact on public health. In response to the emergence of SARS-CoV-2, scientists rapidly developed prophylactic vaccines and began repurposing already available drugs to treat hospitalized patients. In this study, we describe the development of nanoparticles (NPs) using a biocompatible polymer we have developed, poly(glycerol monostearate-co-ε-caprolactone) (PGC-C18), for the pulmonary delivery of antiviral drugs, particularly for the treatment of coronavirus infection. We selected PGC-C18 because of our experience with this polymer in a different drug delivery form factor (i.e., an implantable surgical mesh), its successful completion of 10,993 biocompatibility testing required by the FDA (Kaplan et al, 2016), and its availability via large-scale GMP production processes that would speed translation to the clinic. Furthermore, PGC-C18 exhibits superior long-term compound release properties and lacks an initial drug “burst” release associated with unmodified or short-chain fatty acid–modified PGC surfaces (Wolinsky et al, 2010, 2012).

Other groups have similarly leveraged polymer systems to physically encapsulate antibiotics or antimicrobials for pulmonary delivery (Coowanitwong et al, 2008; Ungaro et al, 2012; Al-Halifa et al, 2019). Polylactic acid (PLA) and poly(lactic-co-glycolic acid) (PLGA) are commonly employed as they are biodegradable and biocompatible polymers present in FDA- and European Medicines Agency (EMA)–approved products for use in humans. However, these formulations tend to form particles ≥200 nm with many manufacturing approaches resulting in microspheres on the μm-size scale. Multiple particle deposition studies suggest that NPs > 100 nm fail to reach deep lung tissue (i.e., the alveoli), which makes these polymer platforms a less attractive approach for prophylactics/treatments against coronaviruses, such as SARS-CoV-2, as this virus largely targets alveolar type 2 (AT2) cells, one of the major cell types that co-express ACE2 and TMPRSS2 (Geiser & Kreyling, 2010; Löndahl et al, 2014; Liu et al, 2021).

We fabricated PGC-NPs using an emulsification and solvent evaporation method, which yielded NPs between 100 and 150 nm in diameter with low polydispersity (<0.17) and low vehicle cytotoxicity. These NPs physically encapsulate small molecule drugs, and the encapsulation efficiency increases with increasing hydrophobicity/lipophilicity (i.e., logP) of the drugs. Among the four drugs loaded—chloroquine, mefloquine, sulfadoxine, and nitazoxanide—mefloquine is the most effectively encapsulated compound with an encapsulation efficiency of ∼63%. MFQ-loaded NPs exhibit controlled release and retain their size and dispersity after nebulization, making them suitable for direct drug delivery into the lung. Furthermore, the NPs exert minimal in vitro cytotoxicity in human lung fibroblast (HFL-1), Vero E6, and Calu-3 cell lines, making them suitable for clinical translation.

Of the investigated drugs, mefloquine shows improved activity against SARS-CoV-2 over other repurposed drug candidates such as hydroxychloroquine and chloroquine, yet remains under-investigated as an antiviral therapy (Shionoya et al, 2021; Sacramento et al, 2022). Jan et al report that orally administered treatment of MFQ at 30 mg/kg/day for 3 d results in the absence of weight loss in SARS-CoV-2–infected hamsters (Jan et al, 2021). However, viral lung titers only decrease by less than one log unit and no further investigation regarding disease progression or lung histology was reported. Preliminary pharmacokinetic modeling suggests that conventional (i.e., oral) dosing of MFQ requires multiple high doses (e.g., 350–450 mg) daily to achieve therapeutic plasma concentration, likely impeding clinical translation of orally dosed MFQ. Alternatively, pulmonary delivery of MFQ would enable higher concentrations in lung tissue, reduce systemic exposure, and mitigate off-target toxicities (i.e., neurotoxicity), while eliminating the need for repeat daily doses. As a first step toward this goal, we designed a polymer-based nanoparticle to deliver mefloquine locally to the respiratory tract, the site of SARS-CoV-2 infection. MFQ-NPs exhibited robust and dose-dependent viral inhibition across multiple coronavirus strains including MHV, HCoV-OC43, and SARS-CoV-2 (USA-WA1/2020 and Omicron BA.1 variants). In addition, unlike hydroxychloroquine and chloroquine, MFQ is efficacious against SARS-CoV-2 infection in cell lines expressing both ACE2 and TMPRSS2 (e.g., Calu-3) (Fig 6 [Shionoya et al, 2021; Sacramento et al, 2022]).

Despite mefloquine being used as an antimalarial drug similar to chloroquine, little is known about the biological response to mefloquine treatment, particularly regarding lysosomal acidification. In acute myeloid leukemia cells, mefloquine disrupts lysosomal integrity while exerting a biphasic effect on lysosomal pH (Sukhai et al, 2013; Lam et al, 2019). Here, treatment with mefloquine or MFQ-NPs decreases lysosomal pH with a concomitant increase in LysoTracker accumulation when compared to non-treated controls. Interestingly, further acidification as a result of mefloquine treatment corresponds to an inhibition of proteolytic degradation at high dosages. Unlike chloroquine, which is a known lysosomotropic agent that increases lysosomal pH, leading to a decrease in lysosomal proteolytic activity, mefloquine inhibits proteolytic activity while exerting the opposite effect on lysosomal pH at therapeutic dosing (Hoffmann et al, 2020). At subtherapeutic dosing in our study, mefloquine treatment decreases lysosomal pH and increases proteolysis, but conversely, lysosomal accumulation increases over baseline. These effects on lysosomal pH and activity resemble a biphasic dose response (hormesis) described previously for chloroquine and its derivatives and may explain the varied outcomes in the treatment of COVID-19 patients with lysosomotropic drugs (Moore, 2020; Calabrese et al, 2021).

In the context of SARS-CoV-2, mefloquine is known to inhibit viral entry, specifically during post-attachment processes (Shionoya et al, 2021; Sacramento et al, 2022). Both in free drug and NP formulations, mefloquine inhibits SARS-CoV-2 entry and replication to a similar degree as E64d and remdesivir post-attachment. However, viral attachment only reduces after transient exposure to virus followed by washing. For both empty NPs and MFQ-NPs, this may be partially explained by steric hindrance of viral attachment because of non-specific adsorption of NPs onto the cell surface. The remarkable effect of E64d inhibiting viral attachment to the cell may be explained by its inhibition of secreted CTSL, which enhances coronavirus entry into cells (Zhao et al, 2021). To further interrogate whether mefloquine exerts any effect on the viral attachment phase, we investigated the expression level of several entry proteins responsible for SARS-CoV-2 uptake in Calu-3 cells and MHV uptake in L929 cells. In response to high-dose (i.e., 17.4 μM) mefloquine in free drug or NP form, RNA transcripts for ACE2 decrease and expression increases for both TMPRSS2 and CTSL. Yao et al report similar results after treatment with hydroxychloroquine in human primary pterygium and conjunctival tissues (Yao et al, 2022). There is some evidence that chloroquine and hydroxychloroquine inhibit ACE2 glycosylation (Brufsky, 2020), which may induce incorrect trafficking of ACE2 and reduce ACE2 expression. As ACE2 is an essential target cell protein for SARS-CoV-2 infection, it is plausible that prolonged (≥48 h) treatment with mefloquine reduces pulmonary cell ACE2 expression, which leads to inhibition of viral attachment in addition to inhibition of post-attachment viral proliferation. The increase in CTSL expression may be the result of a compensatory response to the observed lysosomal accumulation and decrease in lysosomal proteolytic activity after high-dose mefloquine treatments. Remarkably, treatment with hydroxychloroquine slightly increases TMPRSS2 expression levels, indicating a similar response of TMPRSS2 expression regulation to lysosomal inhibition to CTSL (Yao et al, 2022). In L929 cells, murine carcinoembryonic antigen–related cell adhesion molecule (mCEACAM1) expression significantly reduces when treating with high-dose free MFQ or MFQ-NPs. mCEACAM1a is an identified cognate target for MHV spike protein; thus, down-regulation of mCEACAM1 by mefloquine may provide a protective effect against MHV infection. Similarly, a broad reduction in cell adhesion markers including CEACAM1, CEACAM5, CEACAM6, and CEACAM7 occurs in human colon cancer cell lines after treatment with chloroquine (Zamame Ramirez et al, 2020).

Proposed mechanisms for the antiviral effect of mefloquine against coronaviruses and other viruses such as Ebola and monkeypox virus include the inhibition of viral uptake by endocytosis and plasma membrane fusion and release of the viral genome from endosomes; however, a molecular basis for the mechanism is not reported (Shionoya et al, 2021; Sacramento et al, 2022; Akazawa et al, 2023). In fact, MFQ’s effect on endocytosis has not been studied extensively in mammalian cells. In the malaria parasite *Plasmodium falciparum*, mefloquine inhibits the endocytosis of hemoglobin from host erythrocytes without affecting endosome–vacuolar fusion in the parasite suggesting a specific inhibitory effect on the formation of endocytic vesicles (Hoppe et al, 2004). Interestingly in our studies, even at a dosing of 20 μM mefloquine, individual cells stain positive for viral proteins, suggesting that these cells internalize and replicate viral genomes (see Figs 5B, F, and 6B and S4A). However, the surrounding cells do not stain positive even after being in contact with infected cells for periods of time that correspond to multiple SARS-CoV-2 replication cycles. The release/escape of coronaviruses, including SARS-CoV-2, from the cell predominantly relies on exocytosis rather than cell lysis in the first 48 h of infection (Cortese et al, 2020; Calabrese et al, 2021). To that end, coronaviruses hijack autophagic vesicles rather than exocytic vesicles for release (Münz, 2017; Miller et al, 2020; Chen et al, 2021). Mefloquine does not inhibit canonical exocytosis but is a potent inhibitor of autophagic vesicle turnover by inhibiting lysosomal proteases such as cathepsin B (Sharma et al, 2012; Golden et al, 2015; Balasubramanian et al, 2017; Chen et al, 2021). Inhibition of lysosomal protease activity also occurs in our study. This effect varies across cell lines in the literature and was conversely interpreted in some studies as autophagy induction at concentrations that could induce a hormetic compensation (Shin et al, 2015; Xie et al, 2020). We therefore suggest that mefloquine inhibits viral release by blocking both autophagy and viral uptake.

To date, no aerosolized treatment is currently approved against coronaviruses, though local delivery to the lung to treat respiratory viruses is a logical treatment approach (He et al, 2022). Indeed, several groups are exploring aerosolized treatments for COVID-19, with a particular interest in developing remdesivir-containing formulations (Vartak et al, 2021; Ramsey et al, 2022). However, similar to nirmatrelvir and molnupiravir, passage of SARS-CoV-2 WT-WA1 in the presence of remdesivir results in resistance mutations in the RdRp that confer resistance (i.e., 2.7- to 10.4-fold shift in EC_50_) to RDV antiviral activity (Stevens et al, 2022). NPs loaded with mefloquine (MFQ-NPs) exhibit a robust inhibition of coronavirus infection in vitro across multiple viral strains (i.e., MHV-A59, HCoV-OC43, and SARS-CoV-2 WT-WA1 and Omicron BA.1) in cells that model the human pulmonary epithelium (i.e., Calu-3). In summary, we describe a new polymeric nanoparticle system, composed of PGC-C18, which physically encapsulates hydrophobic small molecules with antiviral properties. These results encourage further in vivo investigation and development of MFQ-NPs for use as either a prophylactic or treatment for an array of respiratory coronaviruses.

### Limitations of the study

Several limitations are present in the current study. First, the in vitro effective mefloquine concentration of ca. 17.4 μM required in our viral inhibition assays is slightly higher than in other comparable studies (Shionoya et al, 2021; Sacramento et al, 2022). This result may be a consequence of using 10-100x higher viral MOIs than comparable studies. That said, our MFQ-NPs do not release their entire drug payload in an immediate burst but rather slowly over time and exhibit low cytotoxicity and a potent antiviral effect even under a high viral burden. Our study shows that MFQ-loaded NPs are an effective treatment against SARS-CoV-2 infection. More investigation is needed to determine whether MFQ-NPs release sufficient MFQ in animal lung tissues and whether MFQ-NP treatment is effective in preventing or mitigating SARS-CoV-2 infection in vivo.

## Materials and Methods

### Chemicals

Sodium dodecyl sulfate (L4509), chloroquine diphosphate (C6628), and mefloquine hydrochloride (M2319) were purchased from Sigma-Aldrich. Sulfadoxine (S0899) and nitazoxanide (N1031) were purchased from TCI Chemicals (Tokyo, JP). DQ-Red BSA reagent (D12051), LysoTracker Deep Red (L12492), LysoTracker Green DND-26 (L7526), and LysoSensor Yellow/Blue dextran (L22460) were purchased from Invitrogen. CellTiter 96 AQueous One Solution Cell Proliferation Assay (MTS) and CellTiter-Blue were purchased from Promega. Annexin V-Orange (4759) was purchased from Sartorius.

Antibodies used in this study were against the nucleoprotein of HCoV-OC43, clone 542-7D (MAB9013; Sigma-Aldrich), and against the SARS nucleocapsid protein (200-401-A50; Rockland Immunochemicals, Inc).

Free base chloroquine was prepared from the phosphate salt following previously published procedures (Dodd & Bohle, 2014). Briefly, chloroquine diphosphate salt was dissolved in water in a separatory funnel and sodium hydroxide (1 M) was added until all drugs were precipitated. The precipitate, free base chloroquine, was extracted with dichloromethane, dried over sodium sulfate, filtered, and dried under vacuum overnight.

### Synthesis of PGC-C18 and PGC-C18-Rho

Poly(1,3-glycerol monostearate-co-ε-caprolactone) (PGC-C18) was synthesized following previously published procedures (Wolinsky et al, 2007, 2012). Briefly, ε-caprolactone and 5-benzyloxy-1,3-dioxan-2-one monomers were combined in a Schlenk flask at a molar ratio of 4:1, respectively. This flask was then evacuated and flushed three times with N_2_. The flask was then partially submerged in an oil bath and heated to 140°C. Separately, the tin catalyst (Sn(Oct)_2_, molar ratio of monomer: initiator = 500:1) was added to a separate flask and dried under vacuum for 1 h. Dry toluene was added to the catalyst, and the toluene/catalyst mixture was then injected via a syringe into the monomer-containing flask. The reaction was stirred at 140°C for 48 h until the solution became viscous. The reaction was then removed from heat, and the polymer was dissolved in dichloromethane (DCM) and precipitated in cold methanol three times. The solvent was decanted, and the resulting polymer, poly(5-benzyloxy-1,3-dioxan-2-one-co-ε-caprolactone) (PGC-Bn), was dried under vacuum overnight and isolated as a white solid.

The benzyl-protecting groups were then removed via palladium-catalyzed hydrogenolysis. The resulting mixture was filtered through Celite to remove the palladium on carbon (Pd/C), yielding poly(glycerol-co-ε-caprolactone)(PGC-OH). Poly(glycerol-co-ε-caprolactone) (1 mol eq.), stearic acid (0.3 mol eq.), DCC (0.24 mol eq.), and 4-dimethylaminopyridine (0.1 mol eq.) were dissolved in dichloromethane and stirred at RT for 18 h. The dicyclohexylurea was removed via filtration, and the product, poly(1,3-glycerol monostearate-co-ε-caprolactone) (PGC-C18), was dissolved in dichloromethane (DCM) and precipitated in cold methanol three times. The solvent was decanted, and the final product, PGC-C18, was dried under vacuum overnight. Monomer and polymer structures were characterized by proton (^1^H) nuclear magnetic resonance spectroscopy (NMR) using a Varian INOVA 500 MHz instrument at the Boston University Chemical Instrumentation Center (BU-CIC). All spectra were obtained at ambient temperature with compounds dissolved in CDCl_3_ (7.25 ppm for ^1^H NMR) (Fig S1A and B). PGC-C18 polymer molecular weight and dispersity were determined against polystyrene standards using Agilent 1260 Infinity II GPC-SEC equipped with refractive index and dual-angle light scattering detectors (Fig S1C).

For fluorescent polymer, poly(glycerol-co-ε-caprolactone) (1 mol eq.), rhodamine B (0.2 mol eq.), DCC (0.22 mol eq.), and 4-dimethylaminopyridine (0.1 mol eq.) were dissolved in dichloromethane and stirred at RT for 18 h. Next, stearic acid (0.8 mol eq.) was added and stirred at RT for an additional 18 h. Lastly, the dicyclohexylurea was removed via filtration and the product, poly(glycerol monostearate-co-ε-caprolactone) rhodamine B (PGC-C18-Rho), was dissolved in dichloromethane (DCM) and precipitated in cold methanol three times. The solvent was decanted, and the final product, PGC-C18-Rho, was dried under vacuum overnight.

### Preparation of PGC-NPs

PGC-NPs were fabricated similar to a previously described solvent evaporation approach (Ekladious et al, 2017). The core components, PGC-C18 (200 mg), and free drugs (mefloquine, sulfadoxine, chloroquine, nitazoxanide—25 mg, 12.5 wt%) are dissolved in 2 ml dichloromethane. This solution was placed in a sonication bath for 5 min to quickly form a homogeneous solution. The surfactant, sodium dodecyl sulfate (SDS, 80 mg), is separately solubilized in 10 mM phosphate buffer, pH 7.4 (8 ml). These two solutions were then added via a syringe into a sonochemical reaction vessel and emulsified under an argon blanket in a pulsatile manner (30 min total, 1 s on/2 s off) using Sonics Vibra-Cell VCX-600 Ultrasonic Processor (Sonics & Materials). The resulting nanoemulsion is transferred to a clean glass vial under magnetic stirring for at least 1 h to allow the dichloromethane to evaporate from the NP solution. To ensure the elimination of unassociated SDS, the nanoemulsion is then dialyzed for 24 h against 2 liters of 5 mM phosphate buffer (pH 7.4) using SnakeSkin dialysis tubing (MWCO 10 kD).

Rho-NPs were fabricated similarly, with core consisting mainly of PGC-C18 (140 mg) with a small portion of the fluorescent polymer, PGC-C18-Rho (60 mg). By primarily using the traditional PGC-C18 polymer, nanoparticle structure and size remain unperturbed, yet the particles fluoresce and are visible via microscopy and flow cytometry.

### Scanning electron microscopy

Unloaded NPs and MFQ-NPs were diluted 1:100–1:1,000 times in nanopure water. Aliquots were pipetted onto silicon wafers affixed to aluminum stubs with copper tape and allowed to air-dry overnight. The stubs were then sputter-coated with 5 nm Au/Pd. Samples were then imaged using a Supra 55VP field emission scanning electron microscope (Carl Zeiss AG) with an accelerating voltage of 3–5 kV and a working distance of 6 mm.

### DLS

For sizing measurements, 75 μl of NP solution is diluted in 3 ml nanopure water, and for zeta potential measurements, 30 μl of NP solution is diluted in 1.5 ml 1X PBS. Samples are pipetted into a cuvette, and size and zeta potential are then obtained using Brookhaven NanoBrook Omni (Brookhaven Instruments). All measurements were performed in triplicate (n = 3).

### Quantification of drug loading and release

Small molecule drug loading (e.g., mefloquine, chloroquine, nitazoxanide, sulfadoxine) was measured using high-performance liquid chromatography (HPLC). Serial dilution standards were prepared in a mobile phase composed of 45–0.1% triethylamine/phosphate buffer (pH 3.0) and 55%—acetonitrile. Standard samples for each pharmacologic agent were run for 8 min through a Zorbax SB300-C18 column (150 mm length) and detected through UV absorbance to generate a standard curve. To quantify NP loading, NPs were disrupted by adding acetonitrile to a final volume of 90% (vol/vol). This solution was then re-equilibrated by adding aqueous buffer to match mobile phase composition. This solution was filtered through a 0.22-μm PVDF syringe filter (Millipore) to remove large aggregates or dust before running samples. Samples were run similar to standards.

Mefloquine drug release from MFQ-NPs was measured using UPLC-MS (Waters ACQUITY). Briefly, undiluted MFQ-NPs were loaded into Slide-A-Lyzer MINI Dialysis Devices, 10K MWCO (PI88401), and placed into 14 ml of release buffer (either 1X PBS [pH 7.4] with 1 vol/vol% Tween-20 or 0.1 M acetate buffer [pH 5.0] with 1 vol/vol% Tween-20) in a conical tube. Samples were placed in a 37°C oven equipped with a shaker plate, and MFQ release was measured from the release buffer at 4-, 24-, 48-, 72-, 96-, and 120-h timepoints. UPLC-MS samples were run on Waters ACQUITY UPLC with a binary solvent manager, SQ mass spectrometer, Waters 2996 PDA (photodiode array) detector, and evaporative light scattering detector (ELSD) using an ACQUITY UPLC BEH C18 1.7 μm, 2.1 × 50 mm column (186002350). Serial dilution standards were prepared in a mobile phase composed of 50% release buffer (i.e., 1X PBS [pH 7.4] or 0.1 M acetate buffer [pH 5.0]) and 50% acetonitrile.

### qNano

Particle size and size distribution were also measured using a qNano analyzer (Izon Sciences) coupled with an adjustable nanopore (NP150) and air-based variable pressure module (VPM). MFQ-PGC-NPs and carboxylated polystyrene calibration particles (CPC100; Izon Sciences) were diluted 1:500–1:1,000 times in Tris buffer electrolyte (Izon Sciences) before running samples according to the manufacturer’s protocol. Each recorded measurement consisted of at least 500 particles counted in a 5- to 10-min duration. Particle size and distribution were measured using Izon control suite software.

### Nebulization of NPs

MFQ-NPs were first fabricated as described and diluted to respective concentrations using 10 mM phosphate buffer (pH 7.4) to prevent clogging of the vibrating meshes in the nebulizer at high concentrations. Diluted NPs were pipetted into the top reservoir of an Aerogen Pro nebulizer, and a conical tube was used to collect nebulized vapor. Centrifugation was used to condense the vapor into a liquid solution. Nebulized MFQ-NPs were compared with the prediluted sample, as well as prenebulized dilutions for size and morphological changes using DLS and SEM as described previously.

### Cell culture

Vero E6 cells were obtained from the American Type Culture Collection (CRL-1586; ATCC) and grown in Eagle’s Minimal Essential Medium (Corning) supplemented with 10% FBS and penicillin (100 U/ml)–streptomycin (100 μg/ml) (PenStrep, Gibco). Calu-3 human lung cancer cells were obtained from the ATCC (HTB-55) and grown in EMEM supplemented with 10% FBS and PenStrep. L929 and 17CL-1 mouse fibroblast cells were a kind gift from Volker Thiel’s laboratory at the University of Bern and Elke Mühlberger’s laboratory at Boston University and grown in EMEM supplemented with 10% FBS and Primocin (100 μg/ml) (InvivoGen). HCT-8 cells were obtained from the ATCC (CCL-244) and grown in RPMI 1640 (ATCC) supplemented with 10% horse serum. HFL1 cells were obtained from the ATCC (CCL-153) and grown in F-12K media supplemented with 10% FBS and PenStrep.

### Viruses

SARS-CoV-2 USA-WA1/2020 (referred to as WT-WA1) and Omicron BA.1 were obtained from the Biodefense and Emerging Infections (BEI) Resources of the National Institute of Health (contributed by Mehul Suthar). All work with SARS-CoV-2 was performed at the UCLA high-containment laboratory at biosafety level 3. SARS-CoV-2 was propagated and passaged in Vero E6 cells. HCoV-OC43 was obtained from the ATCC (VR-1558) and propagated in HCT-8 cells. MHV-GFP was a kind gift from Volker Thiel’s lab at the University of Bern and Elke Mühlberger’s lab at Boston University. MHV-A59-GFP was propagated in 17CL-1 cells. Viral titers were determined by assessing viral cytopathic effect (CPE) by microscopy in cells infected with serial 10-fold dilutions, respectively. TCID_50_/ml was calculated using the Reed–Muench method.

### In vitro cell viability

The cytotoxicity of NPs with and without MFQ was evaluated using a tetrazolium-based MTS cell proliferation assay (Promega CellTiter 96 AQueous One Solution Cell Proliferation Assay). HFL1, Calu-3, and Vero E6 were cultured in a 96-well plate at 12,000 cells/well for 1 d, after which the media were exchanged for media containing no-treatment control, unloaded PGC-NPs (1.95, 3.91, 7.81, 15.63, 31.25, 62.5, 125, 250, 500, 1,000 μg/ml NPs), MFQ-NPs (1.95, 3.91, 7.81, 15.63, 31.25, 62.5, 125, 250, 500, 1,000 μg/ml NPs), free MFQ solubilized in DMSO (0.78, 1.56, 3.13, 6.25, 12.5, 25, 50 μM), or DMSO as a vehicle control (0.031, 0.063, 0.125% vol/vol relative to culture media). The cells were then incubated with treatment for 24 h, after which cell viability was quantified with a SpectraMax iD3 (Molecular Devices) plate reader relative to the no-treatment control, after correcting for background absorbance. Alternatively, toxicity was determined using the CellTiter-Blue assay (Promega). Vero E6 or Calu-3 cells were seeded in clear 96-well plates and treated with MFQ-NPs at 1–800 μg/ml, unloaded NPs, or equivalent concentrations of free MFQ solubilized in DMSO for 72 h. Subsequently, CellTiter reagent was added to the wells for 1 h and fluorescence emission was measured at ex:565 nm, em:620 nm using a Tecan Spark 10 M plate reader (Tecan Ltd.).

### Cellular uptake of Rho-PGC-NPs via flow cytometry

FACS analysis of Rho-NP–treated cells was performed with an Attune NxT Flow Cytometer (Invitrogen). HFL1, Calu-3, and Vero E6 were cultured in a 96-well plate at 12,000 cells/well for 1 d, after which the media were exchanged for media containing 75 μg/ml Rho-NPs. The cells were then incubated with treatment for 24 h, after which cells were trypsinized, washed with PBS by centrifugation, resuspended in FACS buffer (PBS + 2% FBS), and then subjected to flow cytometry. Cell debris was excluded by gating on the forward and side scatter plot. The intensity bar graph for uptake is displayed as mean values calculated from median fluorescence intensity for the cell population identified as positive based on the gating strategy.

### Cellular localization of Rho-PGC-NPs via confocal microscopy

HFL1 cells were grown on 12-well glass-bottom plates (P12-1.5H-N; Cellvis) at a density of 50,000 cells/well and grown for 24 h. Cells were then treated with a 75 μg/ml dose of Rho-NPs for 1, 4, or 24 h. Cells were then washed with 1X PBS and incubated with 50 nM LysoTracker Deep Red (Invitrogen) in culture media for 1.5 h. After acidic organelle labeling, cells were washed with 1X PBS and incubated with 1 μg/ml Hoechst 33342 (Thermo Fisher Scientific) and 5 μg/ml Wheat Germ Agglutinin Oregon Green 488 Conjugate (Invitrogen) for 10 min at 37°C. Cells were then washed with 1X PBS, incubated with 1 μg/ml Hoechst 33342 in Live-Cell Imaging Solution (Thermo Fisher Scientific), and imaged immediately on an Olympus FV3000 confocal microscope with a temperature control chamber at 37°C. Cells were imaged using a 60X oil immersion objective. Colocalization of Rho-NPs and LysoTracker dye was determined by calculating Pearson’s coefficient using CellProfiler (Carpenter et al, 2006).

### LysoSensor Yellow/Blue dextran imaging

Vero E6 cells were seeded at a density of 10,000 cells/well into Greiner CellView 4-compartment dishes. After 24 h, cells were stained with 5 mg/ml LysoSensor Yellow/Blue dextran dye in EMEM for 3 h after an overnight medium chase. The next day, cells were incubated for 24 h with 100 μg/ml NPs (±MFQ) or 10 or 20 μM MFQ. Bafilomycin A1 was added at a concentration of 200 nM as an alkalization agent 2–4 h before imaging. Imaging was performed using Zeiss LSM880 (Carl Zeiss AG, Jena, Germany) equipped with a Coherent 2-photon laser at a 2-photon excitation of 720 nm and 2 emission bands at 400–480 nm (blue) and 510–620 nm (yellow) using a 63X oil immersion objective. pH standard curves were generated by permeabilizing LysoSensor Yellow/Blue dextran–stained cells with 10 μM nigericin and 20 μM monensin in pH-clamped buffers ranging from pH 4.5 to 6.0. Lysosomal ROIs and Yellow/Blue staining intensity were determined using CellProfiler.

### LysoTracker Green imaging

Vero E6 cells were seeded at a density of 10,000 cells/well into 96-well Greiner μClear imaging plates. After 24 h, cells were treated with 50–100 μg/ml NPs (±MFQ), 20 μM MFQ, or 200 nM bafilomycin A1 for 24 h and subsequently stained with 1 μM LysoTracker Green and Hoechst 33342. Cells were imaged using Operetta High-Content Imager (PerkinElmer Inc) using a 20X air objective, and lysosomal accumulation was defined as LysoTracker-positive signal area % in expanded nuclear ROIs. Images were analyzed using CellProfiler.

### DQ-Red BSA assay

HFL1 cells were cultured in a 96-well plate at 15,000 cells/well for 24 h, after which the media were exchanged for media containing no-treatment control, empty NPs (12.5, 25, 50, 75, 100 μg/ml NPs), or MFQ-NPs (12.5, 25, 50, 75, 100 μg/ml NPs) for 24 h. Control treatments include bafilomycin A1 (200 nM), and Pepstatin A (10 μg/ml) + E64d (10 μg/ml) for 4 h, or free MFQ (10, 15 μM) for 24 h. After treatment, cells were washed with 1X PBS and incubated with 10 μg/ml DQ-Red BSA reagent (Thermo Fisher Scientific) in culture media for 1 h. After incubation with assay reagent, cells were trypsinized, washed with 1X PBS by centrifugation, resuspended in FACS buffer (PBS + 2% FBS), and then subjected to flow cytometry. Cell debris was excluded by gating on the forward and side scatter plot. Relative protease activity is displayed as mean values calculated from median fluorescence intensity for the cell population identified as positive based on the gating strategy.

### MHV-GFP infection kinetics determination

In order to determine the best timepoint to assess MHV-A49 (MHV-GFP) infection, L929 cells were seeded in Corning 96-well clear-bottom plates and inoculated with MHV-GFP at an MOI of 0.1. A 1:500 dilution of Annexin V-Orange (Sartorius) was added to the cells to monitor MHV-induced cell lysis and cell death. Cells were imaged every hour for 48 h using an OmniFL live-cell analysis platform (CytoSMART Technologies), and growth curves were determined using CytoSMART cloud software.

### NP treatments and viral infections

For MHV-A49 infection and HCoV-OC43 assays, L929 or Vero E6 cells are plated into 96-well Corning imaging plates. 24 h later, cells undergo a 1-h prophylactic pretreatment with NPs ± MFQ (12.5–100 μg/ml), free MFQ (1.25–20 μM), positive control (10 μM remdesivir), or no-treatment control. After pretreatment, media are exchanged with serum-free media containing virus (HCoV-OC43 at an MOI of 1 or MHV at an MOI of 0.1) for 1 h. After 1 h of inoculation, virus-containing media are removed, wells are rinsed with 1X PBS, and treatments are added back to the wells for another 24–48 h. Cells are then fixed with 4% PFA, permeabilized with 0.1% Triton X-100 in PBS, and blocked with 2% BSA and 5% normal donkey serum (NDS), and infection is visualized by immunofluorescence staining of pan-coronavirus nucleocapsid protein for HCoV-OC43 with a mouse monoclonal primary and a donkey anti-mouse Alexa Fluor 488–conjugated secondary antibody and by cell nucleus counterstaining with DAPI. MHV-GFP–positive cells will appear as GFP fluorescence–positive or as syncytia.

For SARS-CoV-2 infection assays, Vero E6 or Calu-3 cells were plated into 96-well clear-bottom imaging plates. 24 h later, cells undergo a 1-h prophylactic pretreatment in 100 μl of media containing 2X concentrated NPs ± MFQ (25–200 μg/ml), free MFQ (2.5–40 μM), positive control (20 μM remdesivir), or no-treatment control. After pretreatment, 100 μl of media containing virus (SARS-CoV-2 at an MOI of 0.1 for Vero E6 and an MOI of 0.2 for Calu-3 cells) is added to the wells diluting the treatments to 1X for another 48 h. Alternatively, for post-inoculation treatments, Calu-3 cells are infected with SARS-CoV-2 at an MOI of 0.2 diluted in serum-free EMEM for 1 h. Subsequently, the inoculum was removed, and the cells were washed once with DPBS, and treated with NPs ± MFQ (12.5–100 μg/ml), free MFQ (1.25–20 μM), positive control (10 μM remdesivir), or no-treatment control for 48 h. Cells are then fixed with 4% PFA, permeabilized with 0.1% Triton X-100 in PBS, and blocked with 2% BSA, 5% NDS, and infection is visualized by immunofluorescence staining of SARS-CoV-2 N protein with a rabbit polyclonal primary and a donkey anti-rabbit Alexa Fluor 568–conjugated secondary antibody and cell nucleus counterstaining with DAPI. Plates were imaged with an Operetta High-Content imager using a 10X air objective, and images were processed using CellProfiler. Positive cells were determined as expanded nucleus ROIs containing above-threshold virus–positive staining, and syncytia were defined as irregularly close nucleus clusters of >40 μm diameter.

### Expression of viral entry proteins

Calu-3 or L929 cells were plated into 12-well plates at 130,000 or 100,000 cells/well, respectively. 24 h later, the media were exchanged for media containing no-treatment control, unloaded PGC-NPs (50, 100 μg/ml NPs), MFQ-NPs (50, 100 μg/ml NPs), free MFQ solubilized in DMSO (8.7, 17.4 μM), or DMSO as a vehicle control (0.044, 0.087% vol/vol relative to culture media). The cells were then incubated with treatment for 48 h, after which RNA was isolated using RNeasy Plus Mini Kit (QIAGEN) according to the manufacturer’s protocol. Total RNA concentration was determined using NanoDrop (Thermo Fisher Scientific), and cDNA was generated using High-Capacity cDNA Reverse Transcription Kit (4368813; Applied Biosystems). TaqMan probes (Thermo Fisher Scientific) were used to measure the expression level of membrane-bound proteins responsible for viral uptake or processing enzymes essential for the production of said transmembrane proteins: hACE2 and hTMPRSS2 in Calu-3 cells. In addition, we measured the expression level of cathepsin L (hCTSL), which is a lysosomal protease essential for S protein processing and endolysosomal membrane fusion in the SARS-CoV-2 endocytic infection route. Similarly, TaqMan probes were used to measure the expression level of mCEACAM1 in L929 samples.

Changes in relative gene expression were quantified using the 2^-∆∆CT^ method, and hGAPDH and mGAPDH were used as housekeeping genes for Calu-3 and L929, respectively.

### SARS-CoV-2 uptake analysis by qRT–PCR

For the SARS-CoV-2 uptake analysis, Vero-E6 cells were seeded into six-well plates at densities of 5 × 10⁵ cells/well and incubated overnight at 37°C with 5% CO₂. The next day, cells were pretreated with varying concentrations of nanoparticles (NPs) or compounds for 1 h in serum-free media (SFM). After pretreatment, cells were exposed to SARS-CoV-2 for 1 h at a multiplicity of infection (MOI) of 0.2 at 4°C (to assess adherence) or at 37°C (to assess endocytosis and replication) in the presence of the respective compounds and NPs in SFM. For adherence-only samples, cells were washed with ice-cold PBS immediately after the infection period and scraped into 500 μl of TRI reagent (T9424; Sigma-Aldrich). For endocytosis and replication samples, cells were washed with warm PBS after the infection period and then incubated in growth media containing the compounds and NPs for 6 and 24 h, respectively, before collection in TRI reagent as previously described. Samples were stored at −80°C until further processing for qRT–PCR.

RNA extraction from cell homogenates was performed using TRI reagent according to the manufacturer’s guidelines. The reverse transcription of RNA to cDNA was carried out using High-Capacity cDNA Reverse Transcription Kit (4368813; Applied Biosystems), following the manufacturer’s instructions. The reverse transcription protocol included an initial 10-min incubation at 25°C, followed by 120 min at 37°C, 5 min at 85°C, and indefinite maintenance at 4°C.

qRT-PCR was conducted using Power SYBR Green PCR Master Mix (4367659; Thermo Fisher Scientific), adhering to the manufacturer’s protocol. The qRT-PCR cycling conditions were an initial 2-min incubation at 50°C, 10 min at 95°C, followed by 40 cycles of 15 s at 95°C and 60 s at 60°C, with SYBR wavelength acquisition. The temperature ramp rate for all cycling steps was set at 1.6°C/second. Each qRT-PCR run included triplicate technical replicates, a 2019-nCOV–positive control plasmid (10006621; IDT), and an infection-negative control sample. Data analysis was performed using the ΔΔCt method for fold change calculation.

### Statistical analysis

Unless otherwise noted, all experiments were repeated at least three times. Unless otherwise mentioned, data were graphed and analyzed using GraphPad Prism 9.1. Data are displayed as means ± SD or SEM where appropriate. To determine statistically significant differences, one-way and two-way ANOVA with Dunnett’s post hoc tests were applied where appropriate.

## Supplementary Material

Reviewer comments

## Data Availability

The raw data required to reproduce these findings are available from the authors upon request.
